# The Spatial Spread and the Persistence of Gene Drives Are Affected by Demographic Feedbacks, Density Dependence and Allee Effects

**DOI:** 10.1111/mec.70028

**Published:** 2025-07-19

**Authors:** Léna Kläy, Léo Girardin, Vincent Calvez, Florence Débarre

**Affiliations:** ^1^ Institute of Ecology and Environmental Sciences Paris (IEES Paris) Sorbonne Université, CNRS, IRD, INRAE, Université Paris Est Creteil, Université de Paris Paris Cedex 5 France; ^2^ Institut Camille Jordan, UMR 5208 CNRS and Universite Claude Bernard Lyon 1 Lyon France

**Keywords:** allee effect, density dependence, gene drive, genetically modified organisms, population dynamics, population genetics—theoretical

## Abstract

Homing gene drive alleles bias their own transmission by converting wild‐type alleles into drive alleles. If introduced in a natural population, they might fix within a relatively small number of generations, even if they are deleterious. No engineered homing gene drive organisms have been released in the wild so far, and modelling is essential to develop a clear understanding of the potential outcomes of such releases. We use deterministic models to investigate how different demographic features affect the spatial spread of a gene drive. Building on previous work, we first consider the effect of the intrinsic population growth rate on drive spread. We confirm that including demographic dynamics can change outcomes compared to a model ignoring changes in population sizes because changes in population density can oppose the spatial spread of a drive. Secondly, we study the consequences of including an Allee effect and find that it makes a population more prone to eradication following drive spread. Finally, we investigate the effects of the fitness component on which density dependence operates (fecundity or survival) and find that it affects the speed of drive invasion in space and can accentuate the consequences of an Allee effect. These results confirm the importance of checking the robustness of model outcomes to changes in the underlying assumptions, especially if models are to be used for gene drive risk assessment.

## 1 Introduction

A promising but controversial new strategy for the control of natural populations, artificial gene drive biases the transmission of particular alleles to the offspring, over expectations of regular Mendelian transmission (Alphey et al. 2020; Burt 2003; Burt and Crisanti 2018). Such alleles can be detrimental to the individuals carrying them, and yet spread in a population thanks to their transmission advantage. Artificial gene drive implementations, so far still restricted to laboratory settings, have achieved transmission rates of 99% in yeast ( *Saccharomyces cerevisiae* ; DiCarlo et al. 2015), more than 90% in mosquitoes ( *Anopheles gambiae* ; Fuchs et al. 2021) and more than 85% in fruit flies ( *Drosophila melanogaster* ; Yang et al. 2022).

In ‘homing drives’, biased inheritance relies on gene conversion: in a heterozygous cell, the gene drive cassette present on one chromosome induces a double‐strand break on a target site on the homologous chromosome and repair by homologous recombination duplicates the cassette. The repetition of this process through generations favours the propagation of the drive allele in the population. Conversion can theoretically happen at different steps of the life cycle, like in the germline of the parents, or in the zygote. Practical implementations in the laboratory have focused on conversion in the germline (Champer et al. 2017).

Biased transmission via gene conversion can lead to the spread of new, potentially deleterious traits in a population within a relatively small number of generations. Two main types of drive can be distinguished: *replacement drives*, aiming to change features of the target population without directly affecting its size, and *suppression drives*, aiming to reduce population size (an extreme being *eradication drives*). Because we are interested in exploring the effect of demographic dynamics on the spatial spread of gene drive alleles, our work here focuses on suppression drives. Experimental proofs of principle for this type of drive have been obtained with cage populations (Kyrou et al. 2018; Hammond et al. 2021), and the feasibility in large populations has been confirmed by theoretical studies (Burt 2003; Godfray et al. 2017; Girardin and Débarre 2021).

Artificial gene drive, such as CRISPR‐based homing drive, holds promise for addressing a number of important real‐world issues (Bier 2022; Hay et al. 2021; Nolan 2020), among which the burden caused by vector‐borne diseases like malaria. Artificial gene drive could be used to spread a new trait rendering progeny of vector mosquitoes unable to transmit disease (Valentino et al. 2015), or simply leading to the reduction of vector mosquitoes population size over time (Kyrou et al. 2018; Hammond et al. 2015). Applications of artificial gene drive are, however, not limited to human health. Gene drive could help conserve or even partially restore native ecosystems by disadvantaging invasive species or favouring endemic ones (Esvelt et al. 2014; Rode et al. 2019). It could also be used in agriculture to reverse insecticide resistance in pest animal species (Kaduskar et al. 2022) or make weeds susceptible again to herbicides (Neve 2018).

At the time of writing, no artificial gene drive organisms have been released in the wild. Laboratory experiments, as well as mathematical and computational models, are crucial to evaluate the risks and benefits of gene drive and to assess the safety of potential releases. Models are, however, simplifications of the living world, and it is crucial to understand the impact and importance of various modelling choices and to test the robustness of results to changes in modelling assumptions.

The simplest theoretical models of gene drive represent well‐mixed populations (Dhole et al. 2020) and focus on allele frequency changes over time (Rode et al. 2019; Deredec et al. 2008; Unckless et al. 2015; de Jong 2017; Tanaka et al. 2017). Here, we investigate the spatial spread of a gene drive allele, and how demographic features affect it.

The spatial spread of an allele has been studied in various contexts for almost a century (Fisher 1937; Kolmogorov et al. 1937). Bistable models and demographic effects, in particular, have been studied in the context of ‘hybrid sinks’, where two viable (sub)species meet and generate unfit hybrids (Bazykin 1969; Barton 1979; Barton and Hewitt 1985; Barton and Turelli 2011; Mallet et al. 1990; Barton 1986). The hybrid zone can move, expanding the area occupied by the fitter (sub)species. The situation we consider here, in the context of gene drive, is however different, because the type expanding its range may be the less fit one, and it may even not be viable on its own. This key biological difference raises a host of new questions that we explore here.

Considering space is important, because being able to increase in frequency in a well‐mixed population does not imply that a gene drive will spread spatially. This is in particular the case when the drive is threshold‐dependent, that is, when, in a well‐mixed population, it needs to be introduced in a high enough amount to increase in proportion (Leftwich et al. 2018; Champer et al. 2016).

A variety of frameworks have been used to study the spatial spread of a gene drive, ranging from generic reaction–diffusion models studied analytically (e.g., Tanaka et al. 2017; Beaghton et al. 2016; Girardin 2019; Kläy et al. 2023) to individual‐based simulations tailored to a specific system and location (e.g., North et al. 2019; North et al. 2020). Each approach has its own limitations: a lack of biological realism sometimes for ones, a lack of generalisability for the others (Levins 1966). While computer simulations allow for a lot of flexibility in model features, in particular when established simulation platforms are used (e.g., Champer et al. 2020, 2021; Pan and Champer 2022; Kim et al. 2023), the simpler mathematical models would often focus on allele frequency changes and neglect demographic dynamics for the sake of analytical tractability. While changes in population density may be ignored when a drive barely affects reproduction or survival, it becomes important to consider them in the case of a suppression drive, because its increase in proportion directly affects population size (Dhole et al. 2020). Previous work on a specific model (Girardin and Débarre 2021; Kläy et al. 2023) found that demographic features can affect the speed of advance of a drive wave over a continuous space. Here, we assess the robustness of this result to different modelling assumptions.

Various demographic features may affect drive spread. A population's growth rate is determined by birth and death rates (Rueffler et al. 2006). Density regulation may affect the two differently, which has consequences for overall demographic dynamics (Tsoularis and Wallace 2002). Likewise, which fitness component is affected by the drive (i.e., whether the drive reduces fecundity or decreases survival) can also influence outcomes (Rode et al. 2020). Finally, growth at low population density may be different from growth at high population densities, that is, Allee effects may operate (Luque et al. 2016). This can be caused by inbreeding depression or difficulties in finding a mate when the population density is low (e.g., Courchamp et al. 2008). Allee effects are frequently observed in the wild, including for animals considered potential targets of control by artificial gene drive, like mosquito species affected by inbreeding depression (Armbruster et al. 2000; Baeshen et al. 2014; Ross et al. 2019). Model outcomes have been shown to be highly dependent on the shape of the density‐dependence function, and in particular on the strength of Allee effects, which can help to achieve suppression or eradication in a targeted population as they lower growth rate at low density (Dhole et al. 2020; May 1973). In two‐sex polygynous mating models, the presence of a strong Allee effect can facilitate the eradication of the targeted population (Wilkins et al. 2018). Similarly, a study on Driving Y chromosomes and homing constructs targeting essential genes has shown that an Allee effect might increase the region of parameter space in which eradication is possible (Beaghton and Burt 2022). In a fragmented population, however, an Allee effect may result in the loss of the gene drive allele before it can spread to other patches (Kim et al. 2023).

In this paper, we study four demographic models considering the presence or absence of an Allee effect, and whether density regulation acts on births or deaths. We extend previous findings from Kläy et al. (2023) and highlight the importance of taking demography into account in the models: the outcomes might potentially shift from a successful spread of a threshold‐dependent drive to its failure. We also show that an Allee effect or a density regulation on the deaths instead of the births might favour the eradication of the targeted population, but also lead to the potential failure of a threshold‐dependent drive. Finally, we prove that a density‐dependence constraint on the deaths instead of the births results in a faster drive invasion. All these findings highlight the importance of ecological details on the outcome of the release of a drive.

## 2 Models and Methods

### 2.1 Models

In this section, we build step by step the different models that we will compare. These models differ in their demographic components, which we first introduce.

#### 2.1.1 Demographic Terms

To assess how sensitive results might be to different demographic modelling choices, we consider four models differing in their birth and death terms. We first illustrate these four demographic models in the case of a genetically and spatially homogeneous population, composed only of wild‐type individuals.

We denote by $n\left(t\right)$ the density of the population at time t, and by r the population's intrinsic growth rate. We will compare density dependence acting on the birth term (Models BN and BA) or death term (Models DN and DA), and the absence (Models BN and DN) and the presence of an Allee effect (Models BA and DA). We denote by a the parameter controlling the Allee effect threshold (when there is an Allee effect, −1≤a≤1).

These four models can be expressed in the following generic form:

(1) $$\partial_{t}n\left(t\right)=\overset{\text{births}}{\overbrace{B\left(n\left(t\right)\right)\;n\left(t\right)}}-\overset{\text{deaths}}{\overbrace{D\left(n\left(t\right)\right)\;n\left(t\right)}}\;\left(\forall t>0\right)$$

The B and D terms in Equation (1) depend on the models that we consider and that we now introduce.

**Model BN:** Density regulation on the birth terms, no Allee effect.

(2a) $$\begin{array}{l} B\left(n\left(t\right)\right)=r\;\left(1-n\left(t\right)\right)+1\\ D\left(n\left(t\right)\right)=1 \end{array}$$

**Model BA:** Density regulation on the birth terms, Allee effect present.

(2b) $$\begin{array}{l} B\left(n\left(t\right)\right)=r\;\left(1-n\left(t\right)\right)\;\left(n\left(t\right)-a\right)+1\\ D\left(n\left(t\right)\right)=1 \end{array}$$

**Model DN:** Density regulation on the death terms, no Allee effect.

(2c) $$\begin{array}{l} B\left(n\left(t\right)\right)=r+1\\ D\left(n\left(t\right)\right)=r\;n\left(t\right)+1 \end{array}$$

**Model DA:** Density regulation on the death terms, Allee effect present.

(2d) $$\begin{array}{l} B\left(n\left(t\right)\right)=r+1\\ D\left(n\left(t\right)\right)=r+1-r\;\left(1-n\left(t\right)\right)\;\left(n\left(t\right)-a\right) \end{array}$$

Not detailed here for simplicity, full formulations of the models ensure that the birth terms $B\left(n\left(t\right)\right)$ are always positive, by setting it equal to 0 if the terms as written in system (2) are ever smaller than 0 (it might happen in Model BA, details in Appendix S1: Section C.2). The death terms $D\left(n\left(t\right)\right)$ are always positive.

In these equations, population density $n\left(t\right)$ is scaled so that the carrying capacity in all models is 1, and time is scaled so that the death rate in the absence of density regulation is 1.

In these models, the population's initial growth rate (i.e., when n→0) is r in the absence of Allee effect, and −ar in the presence of Allee effect. When −1<a<0, the Allee effect is said to be weak (the initial growth rate remains positive), while when 0<a<1, the Allee effect is said to be strong (the initial growth rate is negative; the population only grows if already at high enough density; see Appendix S1: Section B for details).

#### 2.1.2 Drive and Wild‐Type

The demographic models being defined, we now introduce drive and wild‐type genotypes following the same approach as in Girardin and Débarre (2021) and Kläy et al. (2023). We rewrite Equation (1) into a system of three equations, each one describing the dynamics of a genotype: drive homozygotes (DD), heterozygotes (DW) and wild‐type homozygotes (WW). The various models that we consider all have the same form, which is why we use a generic notation with $B\left(n\left(t\right)\right)$ and $D\left(n\left(t\right)\right)$ in system (3) like we did in Equation (1). These terms need to be replaced by their formulas presented in ((2a), (2b), (2c), (2d)) to recover each model.

We denote by $n_{\mathrm{DD}}\left(t\right)$ the density of drive homozygotes at time t, $n_{\mathrm{DW}}\left(t\right)$ the density of heterozygotes, $n_{\mathrm{WW}}\left(t\right)$ the density of wild‐type homozygotes and by $n\left(t\right)=n_{\mathrm{DD}}\left(t\right)+n_{\mathrm{DW}}\left(t\right)+n_{\mathrm{WW}}\left(t\right)$ the total density.

We assume that an individual's genotype affects its fecundity; the birth term therefore differs across genotypes. The drive allele confers a selective disadvantage to the drive to the individual carrying it. Drive homozygotes have a fitness of 1−s, where s is the fitness cost of the drive. Drive heterozygotes have a fitness of 1−sh, where h is the dominance parameter. Wild‐type homozygotes have fitness of 1.

We assume that mating occurs at random: The probability that a pair of parents with given genotypes produces offspring with a specific genotype is detailed in Table S1 (Appendix S1: Section C.1). These probabilities depend on the moment at which gene conversion takes place and on the probability c that gene conversion takes place and is successful (0≤c≤1). Here we assume that gene conversion takes place in the germline, because this is the timing currently successfully implemented in the laboratory (Champer et al. 2017, 2022a). We assume that failed gene conversion leaves the wild‐type allele intact. In other words, we do not consider here the generation of resistance alleles through non‐homologous end‐joining.

With these notations and assumptions, we obtain the following system describing genotype dynamics in our four models:

(3) $$\left\{ \begin{array}{c} \partial_{t}n_{\mathrm{DD}}\left(t\right)=\left(1-s\right)\;B\left(n\left(t\right)\right)\;\frac{\frac{1}{4}\;{\left(1+c\right)}^{2}\;n_{\mathrm{DW}}{\left(t\right)}^{2}+\left(1+c\right)\;n_{\mathrm{DW}}\left(t\right)n_{\mathrm{DD}}\left(t\right)+n_{\mathrm{DD}}{\left(t\right)}^{2}}{n\left(t\right)}\\ -D\left(n\left(t\right)\right)\;n_{\mathrm{DD}}\left(t\right),\\ \partial_{t}n_{\mathrm{DW}}\left(t\right)=\left(1-\mathrm{sh}\right)\;B\left(n\left(t\right)\right)\;\frac{\left(1+c\right)\;n_{\mathrm{WW}}\left(t\right)n_{\mathrm{DW}}\left(t\right)+2\;n_{\mathrm{WW}}\left(t\right)n_{\mathrm{DD}}\left(t\right)+\frac{1}{2}\;\left(1-c^{2}\right)\;n_{\mathrm{DW}}{\left(t\right)}^{2}+\left(1-c\right)\;n_{\mathrm{DW}}\left(t\right)n_{\mathrm{DD}}\left(t\right)}{n\left(t\right)}\\ -D\left(n\left(t\right)\right)\;n_{\mathrm{DW}}\left(t\right),\\ \partial_{t}n_{\mathrm{WW}}\left(t\right)=B\left(n\left(t\right)\right)\;\frac{n_{\mathrm{WW}}{\left(t\right)}^{2}+\left(1-c\right)\;n_{\mathrm{WW}}\left(t\right)n_{\mathrm{DW}}\left(t\right)+\frac{1}{4}\;{\left(1-c\right)}^{2}\;n_{\mathrm{DW}}{\left(t\right)}^{2}}{n\left(t\right)}\\ -D\left(n\left(t\right)\right)\;n_{\mathrm{WW}}\left(t\right) \end{array}\right.$$

#### 2.1.3 Space

Our models so far did not include space; we now add this component, denoted by the variable x. We consider one spatial dimension only for simplicity here. We assume that individuals move in a diffusive manner, and that movement is not affected by genotypes. In other words, all individuals have the same ability to move in space, and they are equally likely to move left or right in one spatial dimension. Movement is represented by the addition of second derivative terms in system (4) ($\partial_{\mathrm{xx}}^{2}$ terms). All genotypes have the same diffusion coefficient (factor in front of the $\partial_{\mathrm{xx}}^{2}$ term) and space is scaled such that this diffusion coefficient is normalised to 1. For mathematical details on how to obtain the second derivative terms, see for instance Okubo and Levin (2002) and Murray (2007).

With these assumptions on space and movement, we obtain the following system of equations for genotype dynamics over space:

(4) $$\left\{ \begin{array}{c} \partial_{t}n_{\mathrm{DD}}\left(t,x\right)=\left(1-s\right)\;B\left(n\left(t,x\right)\right)\;\frac{\frac{1}{4}\;{\left(1+c\right)}^{2}\;n_{\mathrm{DW}}{\left(t,x\right)}^{2}+\left(1+c\right)\;n_{\mathrm{DW}}\left(t,x\right)n_{\mathrm{DD}}\left(t,x\right)+n_{\mathrm{DD}}{\left(t,x\right)}^{2}}{n\left(t,x\right)}\\ -D\left(n\left(t,x\right)\right)\;n_{\mathrm{DD}}\left(t,x\right)+\partial_{\mathrm{xx}}^{2}n_{\mathrm{DD}}\left(t,x\right),\\ \partial_{t}n_{\mathrm{DW}}\left(t,x\right)=\left(1-\mathrm{sh}\right)\;B\left(n\left(t,x\right)\right)\;\frac{\left(1+c\right)\;n_{\mathrm{WW}}\left(t,x\right)n_{\mathrm{DW}}\left(t,x\right)+2\;n_{\mathrm{WW}}\left(t,x\right)n_{\mathrm{DD}}\left(t,x\right)+\frac{1}{2}\;\left(1-c^{2}\right)\;n_{\mathrm{DW}}{\left(t,x\right)}^{2}+\left(1-c\right)\;n_{\mathrm{DW}}\left(t,x\right)n_{\mathrm{DD}}\left(t,x\right)}{n\left(t,x\right)}\\ -D\left(n\left(t,x\right)\right)\;n_{\mathrm{DW}}\left(t,x\right)+\partial_{\mathrm{xx}}^{2}n_{\mathrm{DW}}\left(t,x\right),\\ \partial_{t}n_{\mathrm{WW}}\left(t,x\right)=B\left(n\left(t,x\right)\right)\;\frac{n_{\mathrm{WW}}{\left(t,x\right)}^{2}+\left(1-c\right)\;n_{\mathrm{WW}}\left(t,x\right)n_{\mathrm{DW}}\left(t,x\right)+\frac{1}{4}\;{\left(1-c\right)}^{2}\;n_{\mathrm{DW}}{\left(t,x\right)}^{2}}{n\left(t,x\right)}\\ -D\left(n\left(t,x\right)\right)\;n_{\mathrm{WW}}\left(t,x\right)+\partial_{\mathrm{xx}}^{2}n_{\mathrm{WW}}\left(t,x\right) \end{array}\right.$$

#### 2.1.4 Summary

The systems of the four models are fully written in Appendix S1: Section C, and we summarise all the parameters in Table 1.

**TABLE 1:** Model parameters.

Parameters	Range of values	Description
r	$\left(0,+\infty \right)$	Intrinsic growth rate
c	$\left[0,1\right]$	Conversion rate
s	$\left(0,1\right)$	Fitness cost of drive homozygotes
h	$\left[0,1\right]$	Drive dominance
a	$\left[-1,1\right]$	Allee effect threshold

We focused in this description on genotype densities $n_{\mathrm{DD}}$, $n_{\mathrm{DW}}$ and $n_{\mathrm{WW}}$. Each model can be rewritten to follow total population density n and allele densities $p_{D}$ and $p_{W}=1-p_{D}$ instead (see Appendix S1: Section C.3).

For finite growth rate r, the equations that we obtain for allele frequencies do not have denominators; they are valid for any strength of selection. When the growth rate r is very large, the reaction terms of the models tend to a simpler panmictic model previously introduced by Deredec et al. (2008), with a denominator (for mathematical details, see Appendix S1: Section C.5 and see Girardin and Débarre 2021). This model can then be reduced to a version without denominator when selection is weak (see Figure S3 for the links between the different versions). We consider the whole possible range of r values, and follow not just allele frequencies but also total population density. In this context, weak selection is not required for formulas without denominators.

### 2.2 Numerical Solutions

The models are solved numerically. To do so, we discretised equations in both time and space, and used a Crank–Nicholson method, which is well‐adapted to reaction–diffusion problems (Press et al. 1992). This method is usually stable for large time steps and is second‐order accurate in space and time. In our simulations, the time step is Δt = 1/6 and the final time T=500. The spatial step is Δx = 1 and we adjusted the domain so that the wave is still is the window at the final time. The code is implemented in Python 3.6, with the Spyder environment, and we provide the link to the codes for the simulation in the Data Availability Statement.

### 2.3 Identifying the Different Possible Outcomes

The introduction of drive individuals in a wild‐type population gives rise to a wave of change in genotype densities through space, called a travelling wave (except in the *gene drive clearance* case, see below). Travelling waves propagate with a constant speed, while maintaining their shape in space. We consider an initial condition in which the left half of the domain is full of drive ($n_{\mathrm{DD}}=1$), and the right half is full of wild‐type ($n_{\mathrm{WW}}=1$), as illustrated in Figure S1. In this article, we are not exploring the effect of inoculum size and distribution, which is a question in itself, and arises in particular in the case of threshold‐dependent drives (Barton and Turelli 2011). We therefore choose an initial condition maximising the possibility of drive spread. The model is then solved numerically as described above.

**FIGURE 1:**
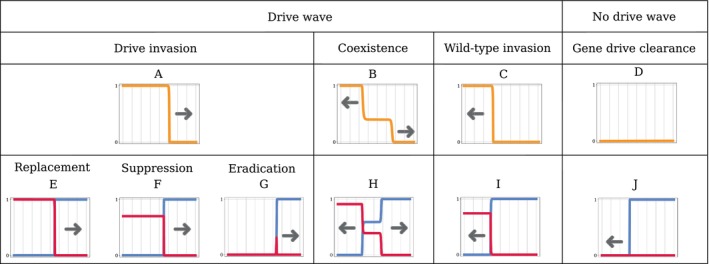
Types of spatial dynamics. Panels A–D show the drive allele frequency. Arrows represent the direction of advance of the wave. Panels E–J show the corresponding allele densities, with the drive allele in red and the wild‐type allele in blue. The horizontal axis represents space.

Following our previous work on a similar model (Kläy et al. 2023), we classify the outcomes into five categories, present in the four models, depending on: the existence or not of a travelling wave; whether the population persists or is eradicated; and in the former case, the genotype(s) present at the end. These outcomes are illustrated in Figure 1.

When the drive travelling wave exists, we distinguish between two cases, depending on the sign of the speed v of the drive wave. When v>0, the wave moves to the right: it is a *drive invasion* (Figure 1A). When v<0, the wave moves to the left: it is a *wild‐type invasion* (Figure 1C). In some specific cases, drive and wild‐type invasions can happen simultaneously: the waves decompose into two sub‐travelling wave solutions over half of the domain. They move in opposite directions and lead to the coexistence of both alleles in‐between (Figure 1B). Finally, there are also instances where the drive wave does not exist at all (Figure 1D), when the drive decays uniformly in space. This happens in particular when a well‐mixed population composed only of drive individuals is not sustainable.

When the drive invades and replaces the wild‐type (Figure 1A), we distinguish three cases depending on the state of the population at the wake of the wave: (i) in the case of *replacement* drives, the population persists in the wake of the wave at the same density as the original wild‐type population (Figure 1E); (ii) in the case of *suppression* drives, the population persists in the wake of the wave, albeit at a lower density than the original wild‐type population (Figure 1F); (iii) in the case of *eradication* drives, the population is eradicated in the wake of the drive invasion wave, leaving empty space behind (Figure 1G).

Finally, when there is no drive wave at all because the drive decays uniformly in space (Figure 1D), the introduced drive sub‐population just dies out, freeing space. The wild‐type population then recolonises the emptied space, at a speed described in the standard Fisher‐KPP travelling wave problem (see Fisher 1937; Kolmogorov et al. 1937; Aronson and Weinberger 1978), that is, in a classical invasion scenario (Figure 1J).

## 3 Results

### 3.1 Demography and Dominance Can Affect the Final Allelic Proportions

Here, we focus on the importance of demography in the model, that is, on the role played by the intrinsic growth rate r on the final allelic proportions. Analytical results can be obtained for r=0 and for r→∞; intermediate cases are investigated numerically.

When r=0, deaths and births compensate each other in a fully wild‐type population. In this limit case, models BN, BA, DN and DA are the same (given in Equation C.5). Both the final densities of all genotypes and the speed of the wave are therefore the same for all models, which we characterise below.

Leaving aside the density‐dependence constraint, the bigger r is, the faster the wild‐type population grows. When r→∞, final allelic proportions are the same in models BN, BA, DN and DA (see Appendix S1: Section C.5). However, the final allelic density and the speed of the wave are not necessarily the same across models for r→∞.

The new result in this part consisted in showing that the four models that we consider share the same final allelic proportion when r=0 and r→+∞. The outcomes are then the same as in the version of the model that we studied in previous work (Kläy et al. 2023), which we recall in this section. From this previous work (Kläy et al. 2023), we introduce the following threshold values of the fitness cost s:

(5) $$s_{1}≔\frac{c}{1-h\left(1-c\right)},\;s_{2,g}≔\frac{c}{2\mathrm{ch}+h\left(1-c\right)}=\frac{c}{h\left(1+c\right)}$$

These threshold values of the fitness cost s determine qualitatively different outcomes. When the drive allele is recessive (h<1/2), $s_{1}<s_{2,g}$; when the drive allele is dominant (h>1/2), $s_{1}>s_{2,g}$.

When the fitness cost s is low enough ($s<\min\left(s_{1},s_{2,g}\right)$), there is a wave of advance of the drive for both r=0 and r→∞ (drive invasion, as in Figure 1A).

When the fitness cost s is high enough ($s>\max\left(s_{1},s_{2,g}\right)$), and the intrinsic growth rate is high (r→∞), the drive wave retreats (wild‐type invasion, as in Figure 1C). When the intrinsic growth rate is low (r=0), $s>\max\left(s_{1},s_{2,g}\right)$ results in drive clearance (as in Figure 1D): the drive is just too costly even for a full‐drive population.

What happens for intermediate fitness cost ($\min\left(s_{1},s_{2,g}\right)<s<\max\left(s_{1},s_{2,g}\right)$) and high growth rate depends on the dominance parameter h. If h<1/2, drive and wild‐type alleles coexist eventually (coexistence, as in Figure 1H). If h>1/2, there is a bistability, the drive is threshold‐dependent: the final outcome is either drive invasion or wild‐type invasion, and depends on the initial conditions.

These results are summarised in Table 2.

**TABLE 2:** Types of model outcomes for Models BN, BA, DN and DA, depending on the fitness cost s, intrinsic growth rate r and dominance parameter h. The outcomes are in terms of allele proportions, as in Figure 1A–D. Model BN was studied in Kläy et al. (2023); we show here that the three other models have the same outcomes when r=0 and when r→∞.

(a) When h<1/2			
	$0<s<s_{1}$	$s_{1}<s<s_{2,g}$	$s_{2,g}<s<1$
r→∞	Drive invasion	Coexistence	Wild‐type invasion
r=0	Drive invasion^a^		Gene drive clearance

(b) When h>1/2			
	$0<s<s_{2,g}$	$s_{2,g}<s<s_{1}$	$s_{1}<s<1$
r→∞	Drive invasion	Bistability	Wild‐type invasion
r=0	Drive invasion^a^	Gene drive clearance	

^a^ The term ‘drive invasion’ here is slightly ambiguous, as it does not specify the genetic composition in the wake of the eradication wave. This exponentially small population might contain the three possible genotypes or only the drive‐homozygous one, depending on parameter choices.

These results illustrate the importance of taking demography into account in the models, notably with the growth rate parameter r. For instance, threshold‐dependent drives (i.e., drives leading to bistabilities) are of particular interest because they are potentially localised and reversible (Tanaka et al. 2017; Leftwich et al. 2018; de Haas et al. 2024). The intrinsic growth rate r is a key component to reach this threshold dependence, as r has to be sufficiently large for the bistability to happen. Indeed, a small r in the range of s values leading to bistability would result in the systematic decay of gene drive alleles (Table 2) and no possibility of drive invasion at all.

As in models without demography nor spatial structure (Deredec et al. 2008; Unckless et al. 2015), the dominance parameter h determines whether threshold dependence can be attained or not: a bistable outcome only exists when $h>\frac{1}{2}$, that is, when the fitness of heterozygous individuals is closer to the fitness of drive homozygous individuals than to that of wild‐type homozygous individuals. This result was already given in a simpler panmictic model (Deredec et al. 2008). Indeed, the panmictic version of models BN and BA (without space, i.e., without the term $\partial_{\mathrm{xx}}p_{D}$) tend to this simpler model for large values of r (See Appendix S1: Section C.5).

### 3.2 The Strength of the Allee Effect and the Type of Density‐Dependence Constraint Affect the Final Allelic Density

In the previous section, we have only described outcomes in terms of allele frequencies. In this section, we compare the final population density $n^{*}$ in the four models, and in particular conditions for which the population goes extinct ($n^{*}=0$). We detail the final densities in all three types of invasions: drive invasion, wild‐type invasion and coexistence. In case of gene drive clearance (decay of the drive allele uniformly in space), the final density is equivalent to the one obtained after a wild‐type invasion: population size goes back to carrying capacity 1.

In all three types of invasion, there are up to three possible regimes: population eradication ($n^{*}=0$); population persistence ($n^{*}=n^{+}>0$); and bistability (the final total population size is either 0 or $n^{+}$ depending on the initial condition relative to a specific density $n^{\tau }$). Note that the bistability involved here is different from the bistability on allele frequencies as seen in the previous section; the bistability that we consider in this section is about population densities. We can write the final population densities in a generic manner for the three types of invasion, as done in Table 3. To do so, we need to define $p_{D}^{*}$, the final proportion of the drive allele in the population. This final proportion is given at equilibrium, that is, setting the reaction terms equal to zero (the first terms of the right‐hand sides of the equations) in the second line of the systems on $\left(n,p_{D}\right)$ detailed in Appendix S1: Section C.4. This leads to the following condition:

(6) $$\left(\left(2h-1\right)\;s\;p_{D}^{*}+\left(1-\mathrm{sh}\right)\left(1+c\right)-1\right)\;p_{D}^{*}\;\left(1-p_{D}^{*}\right)=0$$

**TABLE 3:** Regimes and final densities in Models BN (density regulation on the births; no Allee effect), BA (density regulation on the births; Allee effect), DN (density regulation on the deaths; no Allee effect) and DA (density regulation on the deaths; Allee effect). $p_{D}^{*}$ is the final proportion of the drive allele in the population and $F\left(p_{D}^{*}\right)$ the mean fitness. $n^{+}$ is the final population density when it is greater than 0, and $n^{\tau }$ is a threshold density delimiting basins of attractions of different possible outcomes. We consider up to three regimes regarding the value of the final population density $n^{*}$: Population eradication ($n^{*}=0$), population persistence ($n^{*}=n^{+}>0$) and bistability (the final total population size is either 0 or $n^{+}$ depending on the initial population density relative to the threshold density $n^{\tau }$). The parameter conditions for each of them are given in the ‘Regime’ column.

Model	Regime	$n^{+}$ and $n^{\tau }$ (if it exists)
BN	Eradication if $r<\frac{1-F\left(p_{D}^{*}\right)}{F\left(p_{D}^{*}\right)}$	
	Persistence if $r>\frac{1-F\left(p_{D}^{*}\right)}{F\left(p_{D}^{*}\right)}$	$n^{+\left(\mathrm{BN}\right)}=1-\frac{1-F\left(p_{D}^{*}\right)}{rF\left(p_{D}^{*}\right)}$
BA	Eradication if $r<\frac{1-F\left(p_{D}^{*}\right)}{{\left(\frac{1-a}{2}\right)}^{2}F\left(p_{D}^{*}\right)}$	
	Bistability if $r>\frac{1-F\left(p_{D}^{*}\right)}{{\left(\frac{1-a}{2}\right)}^{2}F\left(p_{D}^{*}\right)}$ and $-\mathrm{ar}<\frac{1-F\left(p_{D}^{*}\right)}{F\left(p_{D}^{*}\right)}$	$n^{\tau \left(\mathrm{BA}\right)}=\frac{1+a-\sqrt{{\left(1+a\right)}^{2}-4\left(a+\frac{1-F\left(p_{D}^{*}\right)}{rF\left(p_{D}^{*}\right)}\right)}}{2}$
	Persistence if $r>\frac{1-F\left(p_{D}^{*}\right)}{{\left(\frac{1-a}{2}\right)}^{2}F\left(p_{D}^{*}\right)}$ and $-\mathrm{ar}>\frac{1-F\left(p_{D}^{*}\right)}{F\left(p_{D}^{*}\right)}$	$n^{+\left(\mathrm{BA}\right)}=\frac{1+a+\sqrt{{\left(1+a\right)}^{2}-4\left(a+\frac{1-F\left(p_{D}^{*}\right)}{rF\left(p_{D}^{*}\right)}\right)}}{2}$
DN	Eradication if $r<\frac{1-F\left(p_{D}^{*}\right)}{F\left(p_{D}^{*}\right)}$	
	Persistence if $r>\frac{1-F\left(p_{D}^{*}\right)}{F\left(p_{D}^{*}\right)}$	$n^{+\left(\mathrm{DN}\right)}=1-\frac{\left(r+1\right)\left(1-F\left(p_{D}^{*}\right)\right)}{r}$
DA	Eradication if $r\left[{\left(\frac{1-a}{2}\right)}^{2}-\left(1-F\left(p_{D}^{*}\right)\right)\right]<1-F\left(p_{D}^{*}\right)$	
	Bistability if $r\left[{\left(\frac{1-a}{2}\right)}^{2}-\left(1-F\left(p_{D}^{*}\right)\right)\right]>1-F\left(p_{D}^{*}\right)$ and $r\left[-a-\left(1-F\left(p_{D}^{*}\right)\right)\right]<1-F\left(p_{D}^{*}\right)$	$n^{\tau \left(\mathrm{DA}\right)}=\frac{1+a-\sqrt{{\left(1+a\right)}^{2}-4\left(a+\frac{\left(r+1\right)\left(1-F\left(p_{D}^{*}\right)\right)}{r}\right)}}{2}$
	Persistence if $r\left[{\left(\frac{1-a}{2}\right)}^{2}-\left(1-F\left(p_{D}^{*}\right)\right)\right]>1-F\left(p_{D}^{*}\right)$ and $r\left[-a-\left(1-F\left(p_{D}^{*}\right)\right)\right]>1-F\left(p_{D}^{*}\right)$	$n^{+\left(\mathrm{DA}\right)}=\frac{1+a+\sqrt{{\left(1+a\right)}^{2}-4\left(a+\frac{\left(r+1\right)\left(1-F\left(p_{D}^{*}\right)\right)}{r}\right)}}{2}$

Each of the three types of invasion corresponds to a value of $p_{D}^{*}$ verifying Equation (6): $p_{D}^{*}=0$ for a wild‐type invasion (i.e., there is no drive in the final population), $p_{D}^{*}=1$ for a drive invasion (i.e., only drive alleles are present in the final population) and

(7) $$p_{D}^{*}=\frac{1-\left(1-\mathrm{sh}\right)\left(1+c\right)}{s\left(2h-1\right)};\;0<p_{D}^{*}<1$$

for coexistence the third possible solution of Equation (6) (i.e., the final population is composed of non‐null frequency of drive and wild‐type alleles).

We also define the mean fitness F:

(8) $$F\left(p_{D}\right)=\left(1-s\right)\;{\left(p_{D}\right)}^{2}+2\;\left(1-\mathrm{sh}\right)\;p_{D}\;\left(1-p_{D}\right)+{\left(1-p_{D}\right)}^{2}$$

Given that F is a monotonic function of $p_{D}^{*}$, we can associate to each type of invasion a value $F\left(p_{D}^{*}\right)$: $F\left(p_{D}^{*}\right)=1$ for wild‐type invasion (setting $p_{D}^{*}=0$ in 8), $F\left(p_{D}^{*}\right)=1-s$ for a drive invasion (setting $p_{D}^{*}=1$ in 8) and $F\left(p_{D}^{*}\right)$ between 1−s and s for coexistence (setting the value of $p_{D}$ given in 7 in Equation 8).

**FIGURE 2:**
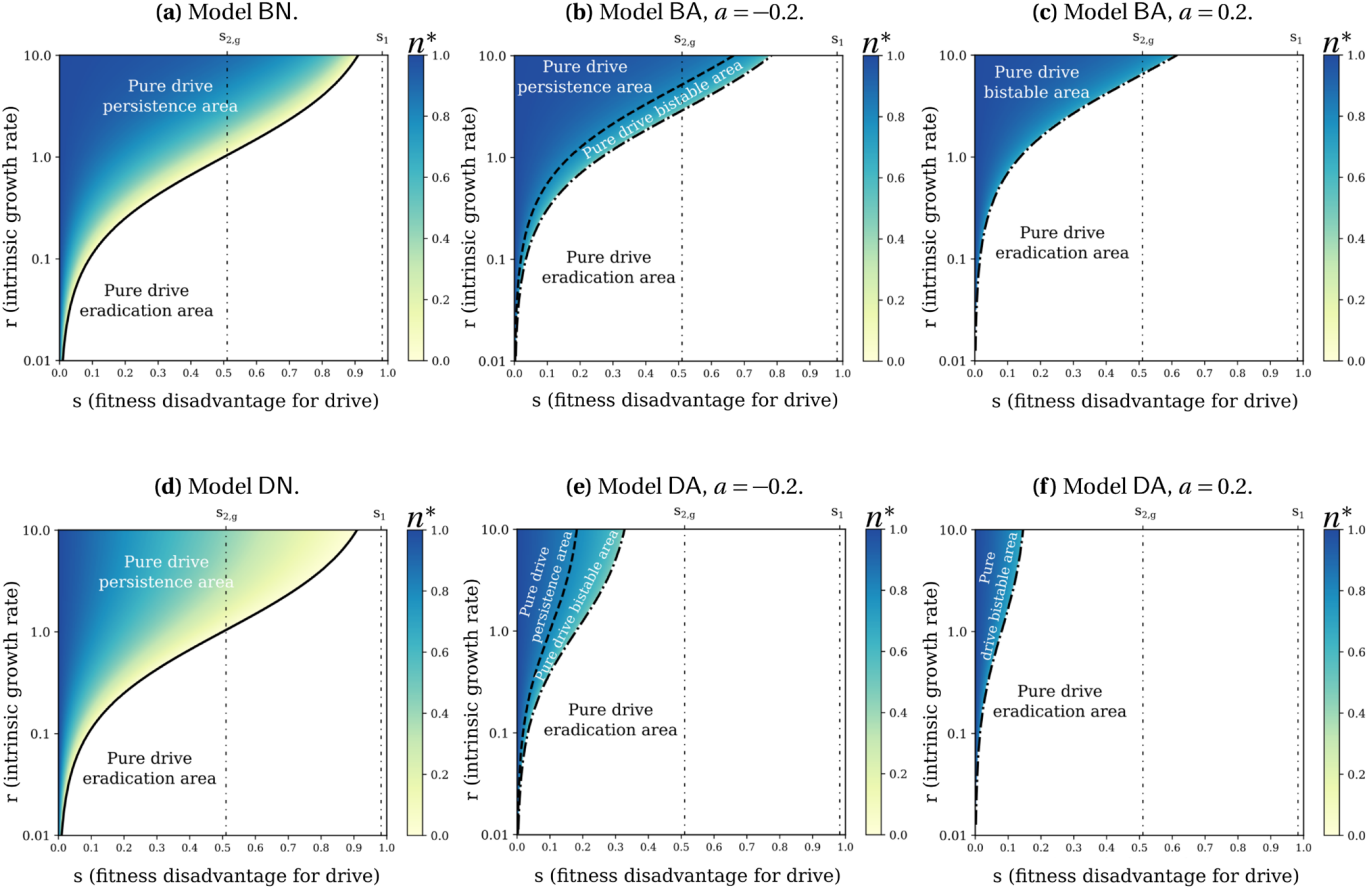
Value of the final population density $n^{*}=\max\left(0,n^{+}\right)$ in case of a drive invasion, shown in shades of colour, with parameters c=0.85 and h=0.9. Since $h>\frac{1}{2}$, drive invasion always occurs for $s<s_{2,g}$, is not systematic for $s_{2,g}<s<s_{1}$, and never occurs for $s_{1}<s$ (see Table 3). The ‘pure drive’ areas correspond to the final population density expected in case of a drive invasion: persistence ($n^{*}=n^{+}$), eradication ($n^{*}=0$) or bistability (either $n^{*}=n^{+}$ or $n^{*}=0$ depending on the initial condition). These final densities are given in Table 3 with $p_{D}^{*}=1$, because the proportion of drive alleles in the final population is one in case of a drive invasion without coexistence.

**FIGURE 3:**
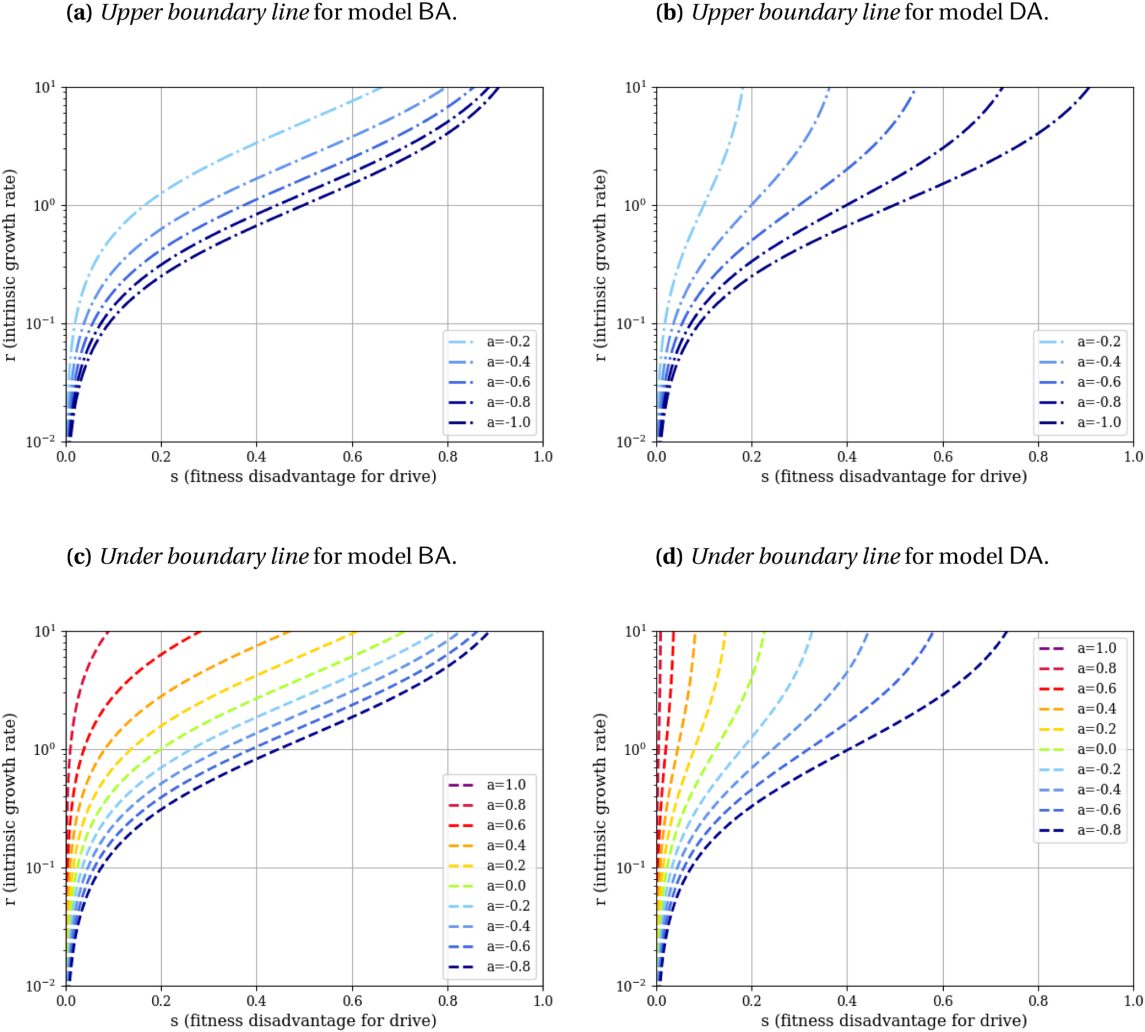
Boundary lines delimiting areas of population persistence, bistability or eradication, under the hypothesis of a drive invasion; we denote these areas ‘pure drive persistence area’, ‘pure drive bistable area’ and ‘pure drive eradication area’ respectively. These boundary lines are drawn for different values of a in models with Allee effect BA and DA. The *upper boundary line* plotted on panels (a) and (b), delimits the pure drive persistence area (above it) from the pure drive bistable area (under it). It is represented with a dash‐dotted line. The *under boundary line* plotted on panels (c) and (d), delimits the pure drive bistable area (above it) from the pure drive eradication area (under it). It is represented with a dashed line.

The final densities $n^{*}$ are computed by solving the allelic frequency systems on $\left(n,p_{D}\right)$ (which become $\left(n^{*},p_{D}^{*}\right)$ at equilibrium) from Appendix S1: Section C.4 with the relevant value of $p_{D}^{*}$. These results, holding for all values of the intrinsic growth rate r, are detailed in Table 3 and illustrated in Figure 2 in case of a drive invasion with c=0.85 and h=0.9. Up to two boundary lines are delimiting areas of population persistence, bistability or population eradication (under the hypothesis of a drive invasion; we denote these areas ‘pure drive persistence area’, ‘pure drive bistable area’ and ‘pure drive eradication area’ respectively). The variation in the boundary lines according to the strength of the Allee effect a (in Models BA and DA) are plotted in Figure 3.

#### 3.2.1 When the Allee Effect Gets Stronger, the Population Is More Prone to Eradication

In the models without Allee effects, there is no bistability for the final population size once the type of invasion is known (see Table 3). However, and as seen before, the type of invasion itself might change depending on the initial conditions (bistability on allele frequency, see Table 2b), and consequently can still affect the final population size. The situation is different in models with Allee effect, where the final population size might depend on the initial conditions. In case of a weak Allee effect (−1<a<0), the three possible regimes are eradication, persistence and bistability. In case of a strong Allee effect (0<a<1), the only two possible regimes are eradication and bistability.

The condition for eradication is the same in all four models if we set a=−1. However, as a grows, that is, when the Allee effect gets stronger, the ranges of s (fitness disadvantage for drive) and r (intrinsic growth rate) leading to population eradication become larger in the models with Allee effect BA and DA (Figure 3, see calculus in Appendix S1: Section D).

Similarly, when a grows, the ranges of s and r leading to population persistence become smaller in Models BA and DA (Figure 3, see calculus in Appendix S1: Section E). We observe that the larger a is, the more the ‘persistence’ and ‘bistable’ regimes are restricted to high values of r and small values of s (Figure 3). In the case of a strong Allee effect (0<a<1), the ‘persistence’ regime even disappears (Figure 3, details in Appendix S1: Section E).

If the drive persists in the final population, then, given how our models are formulated, the stronger the Allee effect, the smaller the final population size in Models BA and DA (Appendix S1: Section F.2).

#### 3.2.2 A Density‐Dependence Constraint on the Deaths Instead of the Births Might Favour Eradication and Reduce the Final Allelic Density in Case of Drive Persistence

The conditions for eradication or persistence are the same in Models BN and DN, that is, they do not depend on whether the density dependence acts on the births or deaths (no ‘bistable’ regime for these models, Table 3 and Figure 2). However if we consider the models with Allee effect for a given a value, there is a greater range of parameters leading to eradication when density dependence acts on the deaths (Model DA) than when it acts on the births (Model BA) (Figure 2, calculus in Appendix S1: Section D). Interestingly, when r→∞, eradication is still possible in Model DA while it is not in Model BA (Figure 2, calculus in Appendix S1: Section D).

If the drive persists in the final population, the final population size is lower in models without Allee effect when the density‐dependence constraint acts on the death rate (Model DN) than on the birth rate (Model BN, see Appendix S1: Section F.1). The same conclusion holds for models with Allee effect: for a given a value, if the drive persists, the final population is less dense in Model DA than in Model BA (Appendix S1: Section F.2).

### 3.3 A Density‐Dependence Constraint on the Deaths Instead of the Births Results in a Faster Invasion

We now focus on the speed of a drive invasion, that is, the speed of the travelling wave emerging from a drive invasion (see Section 2.3).

A speed v of the wave can be calculated when the models are simplified (linearised) assuming low drive density. This speed corresponds to the speed of drive invasion when the movement of individuals is caused by the few drive individuals at the expansion edge, where the drive density is low. In this case, the wave is called a ‘pulled wave’. This happens when such small populations have high growth rates, because the movement is then mainly driven by new births. When movement is brought about by individuals in the bulk of the wave (i.e., in the case of a ‘pushed wave’), the calculated speed corresponds to a lower estimate of the speed: the real speed is higher, but cannot be calculated in general. In a previous article (Kläy et al. 2023), we showed that the calculated speed v corresponds to the speed of a drive invasion when the dominance parameter h is lower than $\frac{1}{2}$, or for a drive fitness cost s small enough when $h>\frac{1}{2}$ (for a precise condition, see Kläy et al. 2023). This result was rigorously proven for large and small values of the intrinsic growth rate r, and numerically observed for all r. We calculate and compare this speed value in our four models (mathematical details are given in Appendix S1: Section C.6).

In models BN and BA with density dependence acting on the birth term, this speed is given by:

(9) $$v^{\left(B\right)}=2\sqrt{\left(1-\mathrm{sh}\right)\;\left(1+c\right)-1}$$

In models DN and DA with density dependence acting on the death term, the speed becomes:

(10) $$v^{\left(D\right)}=2\sqrt{\left(1+r\right)\;\left[\left(1-\mathrm{sh}\right)\;\left(1+c\right)-1\right]}$$

The speeds only exist for $\left(1-\mathrm{sh}\right)\left(1+c\right)>1$ (or equivalently $s<s_{2}$, with $s_{2,g}$ given in Equation 5), which is the necessary condition to have a strictly positive drive allele production at the front of the wave. To understand why, first note that the density of drive alleles is very low at the front of the wave. Therefore, we can make the approximation that at least one parent in each couple formed at the front of the wave is a wild‐type homozygote WW. Consequently, the offspring carrying a drive allele are necessarily heterozygotes: in the front of the wave, the production of drive alleles only relies on the heterozygotes. These heterozygotes have a fitness of $\left(1-\mathrm{sh}\right)$ and produce drive alleles at rate $\left(1+c\right)$: therefore, for a drive invasion to be possible, the production rate $\left(1-\mathrm{sh}\right)\left(1+c\right)$ of drive alleles should be above the rate 1 at which they disappear. The higher the production rate is, the faster the wave moves.

That the wild type is at carrying capacity at the front of the wave also explains why the speeds given in Equations (9) and (10) do not depend on Allee effects: the total density at the front of the wave is already above the Allee threshold.

**FIGURE 4:**
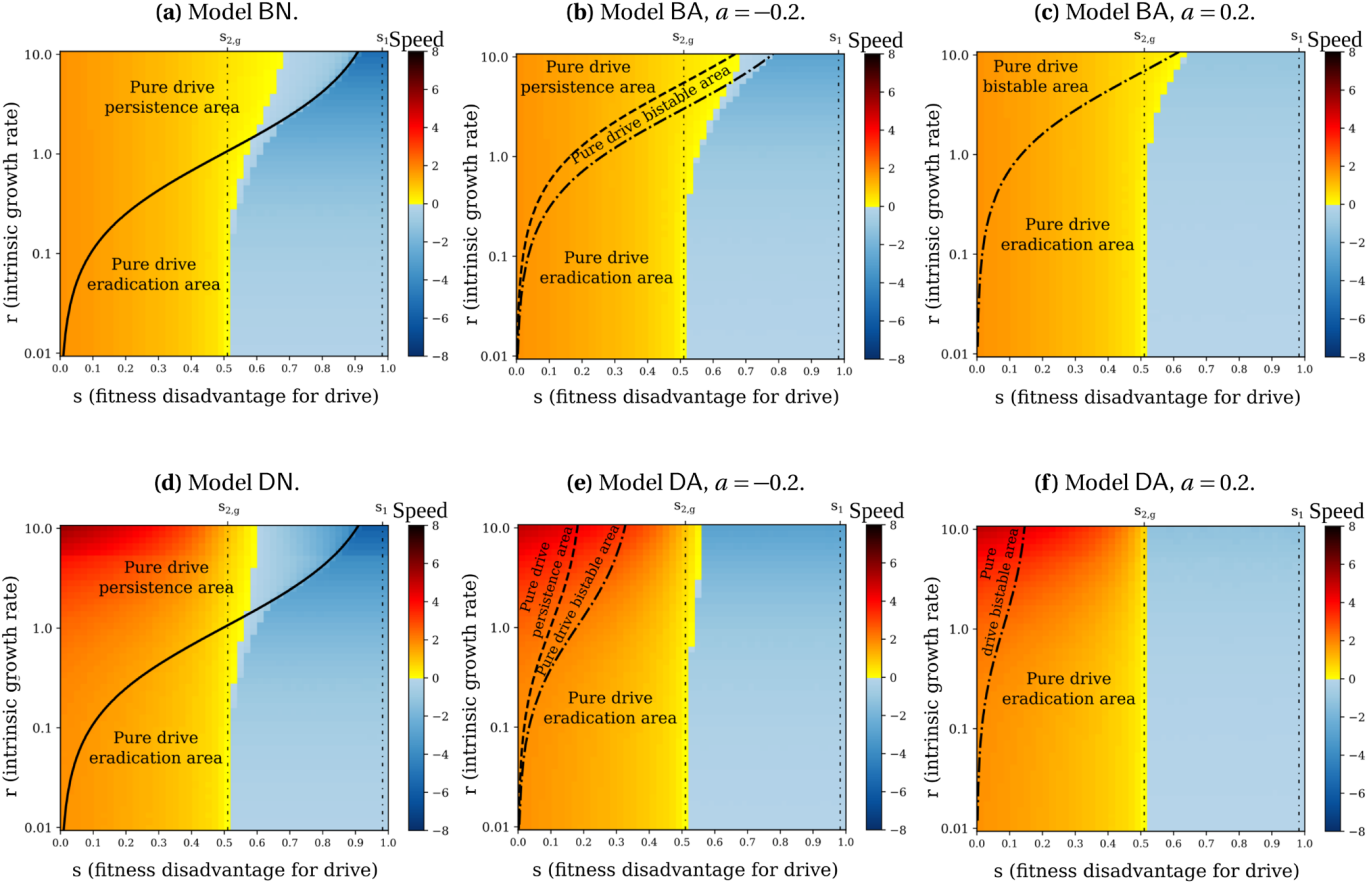
Speed of the wave of drive alleles shown as shades of colour, with parameters c=0.85 and h=0.9. When the drive invades the population, the speed is positive (in yellow‐orange). On the contrary, when the wild‐type invades the population, the speed is negative (in blue). The ‘pure drive’ areas correspond to the final population density expected in case of a drive invasion: persistence ($n^{*}=n^{+}$), eradication ($n^{*}=0$) or bistability (either $n^{*}=n^{+}$ or $n^{*}=0$ depending on the initial condition). Since $h>\frac{1}{2}$, coexistence of drive and wild‐type alleles in the final population is impossible (see Table 2b). Each model is initiated with the initial conditions described in Figure S1. For $s_{2,g}<s<s_{1}$, the speed value may vary if we consider different initial conditions (bistability on the final allele frequencies, see Table 2b). On the left of the vertical line $s=s_{2,g}$, the speed value is independent of r when the density‐dependence constraint acts on the births (Models BN and BA) while it grows with r when the density‐dependence constraint acts on the deaths (Models DN and DA).

The speeds $v^{\left(B\right)}$ and $v^{\left(D\right)}$ are very similar but differ by one coefficient: $v^{\left(D\right)}$ is $\sqrt{r+1}$ times larger than $v^{\left(B\right)}$. This difference relies on the density‐dependence constraint, affecting either the births or the deaths. At the front of the wave, the population density, composed nearly only of wild‐type individuals, reaches the maximum carrying capacity. Consequently, the density‐dependence constraint hinders any increase in the population density, and this happens in two different ways: in models BN and BA, density dependence limits the births so that they do not exceed the deaths, whereas in models DN and DA, it increases the death rate to compensate the births. As a result, the turnover rate is greater in models DN and DA, which induces a faster invasion because the wave movement is mainly driven by new births. Details of the speed calculations are given in Appendix S1: Section C.6. To illustrate this result, we plot the speed of the wave for the four models in Figure 4 and observe that the speed of the drive invasion always increases with r in models DN and DA, in contrast with models BN and BA, for which the speed of the wave does not depend on r. Note however that while the speed $v^{\left(B\right)}$ is independent of r for a small enough drive fitness cost s, it is not the case for the final density $n_{D}^{*}$, which depends on r (Table 3 and Figure 2, models BN and BA). As a result, for a small enough s in models BN and BA, the wave travels at a constant speed no matter the density of population left behind.

### 3.4 A Stronger Allee Effect or a Density‐Dependence Constraint on the Deaths Instead of Births Might Lead to the Failure of a Threshold‐Dependent Drive

Since we set the drive dominance h=0.9, drive invasions are non threshold‐dependent for a fitness cost s smaller than $s_{2,g}$ and threshold‐dependent for a fitness cost s in between $s_{2,g}$ and $s_{1}$ (Table 2b). This is illustrated in Figure 4, where the region corresponding to s smaller than $s_{2,g}$ appears entirely yellow‐orange, indicating that the drive always invades, whereas for s in between $s_{2,g}$ and $s_{1}$, the region is partially blue and partially yellow‐orange, indicating that the drive invasion occurs only if it is introduced in sufficiently large quantities.

The Allee effect seems to reduce the range of parameters leading to drive invasion in threshold‐dependent cases: for $s_{2,g}<s<s_{1}$, some yellow‐orange areas in heatmaps (a) (resp. (d)) turn blue in heatmaps (b) and (c) (resp. (e) and (f)) (Figure 4). This effect becomes more pronounced as the Allee effect get stronger (for larger values of a). Similarly, density regulation on the deaths instead of the births also tends to reduce the range of parameters leading to drive invasion in threshold‐dependent cases.

## 4 Discussion

Understanding the conditions for the spatial spread of an artificial gene drive and its consequences on a targeted population is essential before considering any field release. Laboratories experiments provide information on gene drive dynamics in a small confined and controlled environment, and mathematical models can help gain further insights at small and larger scales.

Theoretical models are meant to provide insights on real‐world dynamics, so it is important to assess how the result of a model depends on modelling choices. In this article, we investigated the influence of considering (i) demography, and more precisely different values of the intrinsic growth rate of the target population, (ii) the presence/absence of an Allee effect and (iii) which fitness component (birth or death) is affected by density dependence. We considered the effects of these features on the type of outcome, on final population density and on the speed of the wave. We considered a one‐dimensional continuous environment, and we studied the spatial spread (or failure) of a drive allele invading an established wild‐type population. We followed the densities of the different genotypes (drive homozygous, wild‐type homozygous and heterozygous) over space and time using partial differential equations. We compared four types of demographic models, depending on the presence or absence of an Allee effect, and the fitness component (birth or death) on which density dependence operates. We characterise the spread of a drive by the existence and direction of its wave of advance, by the final total population density after the drive has spread (or failed to), and by the speed of the wave.

We first described the different qualitative outcomes, extending results from our previous studies (Girardin and Débarre 2021; Kläy et al. 2023) on the importance of taking into account demography in the models. We confirm that the intrinsic growth rate r qualitatively affects results at intermediate values of the fitness cost s. A high intrinsic growth rate leads to a threshold‐dependent drive invasion, while a low intrinsic growth rate results in the decay of drive alleles uniformly in space. Models not considering population densities but focusing on frequencies (e.g., Rode et al. 2019; Deredec et al. 2008; Unckless et al. 2015; de Jong 2017; Tanaka et al. 2017) have dynamics similar to our models provided r→∞. However, they might lead to incorrect conclusions if the intrinsic growth rate of the population is low in reality: for intermediate values of the drive fitness cost for instance, they might predict a successful invasion of a threshold‐dependent drive when the invasion would fail in reality.

As intuitively expected and in agreement with previous results (Wilkins et al. 2018; Beaghton and Burt 2022), introducing an Allee effect in our models makes the population more susceptible to eradication, widening the range of s (fitness disadvantage for drive) and r (intrinsic growth rate) values leading to population eradication after a drive invasion. This phenomenon is accentuated when the Allee effect gets stronger (for larger values of a). In addition, given the way our models are formulated, in models with Allee effect, the larger a, the lower the final population density in case of drive persistence, meaning that an Allee effect might represent a non‐negligible helping force to eradicate or suppress natural populations.

We also considered in our models the impact of whether the density‐dependence constraint targets births or deaths: close to the maximal carrying capacity, in case of rarefaction of the resources, the net growth of the population is limited by either a low number of offspring per generation or a high death rate. We show that when density dependence acts on the deaths, it acts in concert with the Allee effect by enlarging the eradication conditions and reducing the final density, compared to when density dependence acts on the births. How density dependence acts also strongly impacts the speed of propagation: a drive invasion would be $\sqrt{r+1}$ times faster for a density‐dependence constraint on the death rate instead of the birth rate. This prediction holds for a fitness cost reducing the birth rate (individuals carrying drive alleles have fewer offspring than wild‐type ones). However, the conclusions might change for a fitness cost increasing the death rate instead, as shown in a different model of CRISPR‐based homing drives (Rode et al. 2020).

We also showed that an Allee effect might reduce the range of parameters leading to a threshold‐dependent drive invasion. This influence is accentuated when the Allee effect gets stronger (for larger values of a). Similarly, density regulation on the deaths instead of the births also tends to reduce the range of parameters leading to threshold‐dependent drive invasion. Threshold‐dependent drives are of particular interest as they might potentially be localised—though not always (de Haas et al. 2024).

**FIGURE 5:**
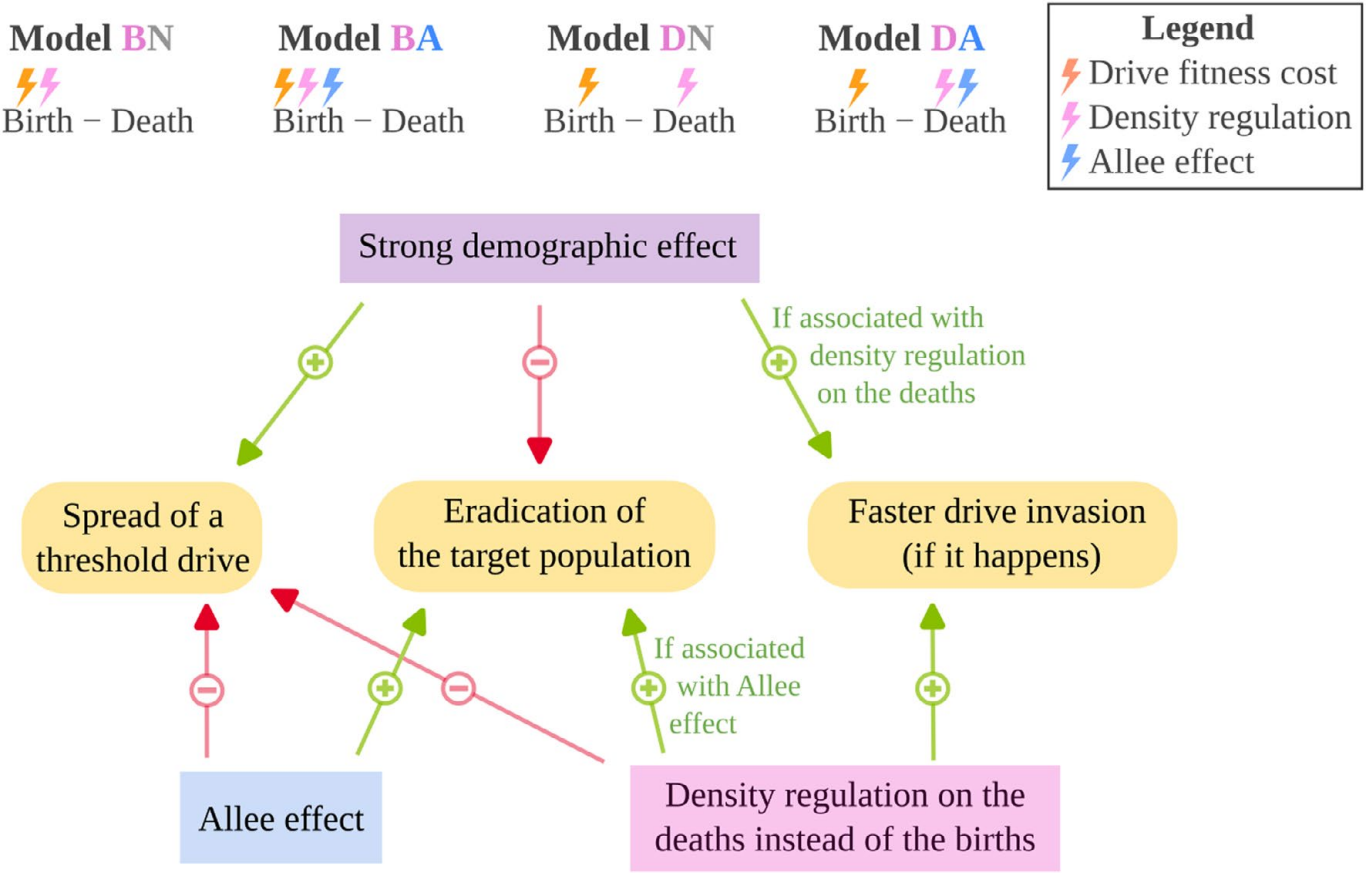
Graphical abstract presenting the four models studied and the main results of this article. The rectangles correspond to features that are included in our models with demography, that is, following both allelic frequencies and densities, compared to simpler models following only allelic frequencies (e.g., Deredec et al. 2008). These two types of models differ most when demographic effects are strong, which in our models corresponds to a small intrinsic growth rate.

The main results of the paper are synthesised in Figure 5.

Our models are deterministic and represent space as continuous and homogeneous. This deterministic, spatially continuous framework can describe population dynamics at large scales, but cannot capture ‘chasing’ events, whereby wild‐type individuals recolonise empty space in the wake of the wave of an eradication drive, and which can arise at low population densities (North et al. 2019, 2020, 2013; Champer et al. 2021; Eckhoff et al. 2017). Considering discrete individuals, and therefore including stochasticity, may lead to qualitatively different outcomes (Durrett and Levin 1994), like the recolonisation by wild‐type individuals of emptied space (Champer et al. 2021; Kläy et al. 2025). Such stochastic fluctuations are likely to be important in particular in the case of suppression and eradication drives, and are left for future investigation. Discrete spatial structures, with small numbers of sub‐populations, may also lead to chasing‐like behaviour at the transition between regimes in a deterministic discrete‐time model (Harris and Greenbaum 2023). Previous work has shown that the conclusions we obtained for a one‐dimensional environment may qualitatively extend to two‐dimensional environments (Tanaka et al. 2017). However, the type of model we used ignores real‐life spatial heterogeneities that may affect the spatial spread of an allele (Dhole et al. 2020; Kim et al. 2023; Piálek and Barton 1997) and heterogeneities in population distribution that may even stop a wave that spreads in a continuous environment (Barton 1979; de Haas et al. 2024; Keitt et al. 2001).

Among the deterministic models in the literature, the models we develop are generalist: they could be applied to different species and any gene drive construct reducing the fitness of the individual carrying it. These models do not aim to bring precise and quantitative predictions, for which more specific models need to be developed, but rather get some insights into the possible outcomes and dissect the roles played by different model elements. However, this generalist approach naturally comes with simplifications, and real‐life applications would need to use models adapted to the specific biological features of the studied system.

In our models, we assume that gene conversion either successfully takes place or does not take place. We did not include resistance alleles which can emerge when conversion fails and repairs by non‐homologous end‐joining occur, or resistance due to standing genetic variation at the target locus. The emergence of resistance alleles can alter the propagation of the drive, but can be mitigated by specific constructs (Rode et al. 2019; Beaghton et al. 2019; Hammond et al. 2017; Price et al. 2020). It is also possible that the speed at which an eradication drive spreads may affect the chances of the emergence and spread of resistance alleles, as in models of evolutionary rescue (Marrec and Bitbol 2020).

Some other simplifications are directly related to the biological characteristics of the species. The polyandrous mating system of mice populations can limit the spread of gene drives (Manser et al. 2019, 2020) or mate search capabilities (Birand et al. 2022). In mosquito populations, the plural life stages (egg, larva, pupa and adults) might influence the modelling conclusions and need to be taken into account by including corresponding age structure in models (Marshall and Hay 2014; Sánchez C. et al. 2020; Champer et al. 2022b). In bee populations, the haploid phases of the life cycle result in less powerful drives: the conditions for fixation are narrower and the spread is slower (Liu et al. 2023; Li et al. 2020). Finally, it is not rare that males and females have different fitness values in transgenic mosquitoes (Kyrou et al. 2018; Hammond et al. 2015; Beaghton et al. 2016; North et al. 2020): more specific models than ours would need to include sex differences.

Finally and more broadly, species do not live in isolation, and interactions of the targeted species within its ecosystem would need to be considered. Competing species or predators can facilitate drive‐based suppression (Liu et al. 2023), and environmental conditions such as seasonality (dry or wet season) can highly impact the eradication of mosquito populations (e.g., North et al. 2019, 2020; Eckhoff et al. 2017). It is of public utility to also consider the impact of gene drive on the whole ecosystem and anticipate the potential risks: the probability of transmission of the gene drive cassette to another species (Connolly et al. 2023), or the cascade of population dynamics and evolutionary processes potentially initiated by the eradication of a species (National Academies of Sciences, Engineering, and Medicine 2016).

Overall, we have shown the importance of considering precise population dynamics on the outcome of the release of a drive. This approach through theoretical models gives first interesting insights that now need to be enhanced with ecological knowledge on specific systems.

## Author Contributions

Léna Kläy and Florence Débarre designed the research, Léna Kläy performed the research with input from all authors, Léna Kläy wrote the article with inputs from Florence Débarre, and all authors edited the article.

## Conflicts of Interest

The authors declare no conflicts of interest.

## Supporting information


Appendix S1.

## Data Availability

The code for the simulations is available on GitHub: https://github.com/LenaKlay/The‐spatial‐spread‐of‐gene‐drives. We ran our simulations in Python 3.6, with the Spyder environment. Heatmaps in Figure 4 were computed using the INRAE Migale bioinformatics facility.
