# Supplementary material for: The Spatial Spread and the Persistence of Gene Drives Are Affected by Demographic Feedbacks, Density Dependence and Allee Effects
Source: Mol Ecol. 2025 Jul 19;34(16):e70028. doi: 10.1111/mec.70028 (PMC12329647; doi:10.1111/mec.70028)
Supplement: Supplementary file 1 — Appendix S1. [file MEC-34-e70028-s001.pdf]

Appendix

A Initial conditions

All our simulations in one-dimensional space are initiated with the initial conditions described in the main text, and illustrated in Figure S1:

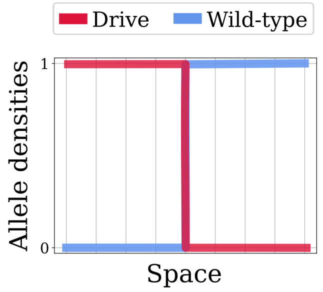

Figure S1: Initial conditions used in the simulations. The left half of the domain is full of drive ($n_{DD} = n_D = 1$), and the right half is full of wild-type ($n_{WW} = n_W = 1$).

B Allee effect

We consider the equation describing the dynamics of the population density n:

$$\partial_t n - \partial_{xx}^2 n = n\,(1-n)\,\sigma(n) = f(n) \qquad \text{with } n \in [0,1]. \tag{B.1}$$

The Allee effect characterises a positive correlation between population density and the per capita population growth rate.

Without Allee effect, the population growth rate $f(n)$ is always positive and the per capita population growth rate $\frac{f(n)}{n}$ is maximum as the population density n tends to zero. This happens for example when $\sigma(n) = 1$ in Equation (B.1) (Figure S2a). Mathematically, we write:

$$\max_{n \in]0,1]} \left(\frac{f(n)}{n}\right) \leq f'(0). \tag{B.2}$$

With a weak Allee effect, the population growth rate $f(n)$ is still positive, but the maximum of the per capita population growth rate $\frac{f(n)}{n}$ is reached at a strictly positive population density. This happens for example when $\sigma(n) = (n-a)$ with $-1 < a < 0$ in Equation (B.1) (Figure S2b). Mathematically:

$$\max_{n \in]0,1]} \left(\frac{f(n)}{n}\right) > f'(0) > 0. \tag{B.3}$$

Finally, with a strong Allee effect, the population growth rate $f(n)$ is negative for small population density, and positive after. This happens for example when $\sigma(n) = (n-a)$ with $0 < a < 1$ in Equation (B.1) (Figure S2c). Mathematically:

$$\exists\, a > 0 \text{ such that } \forall n \in]0,a[\;\; \frac{f(n)}{n} < 0. \tag{B.4}$$

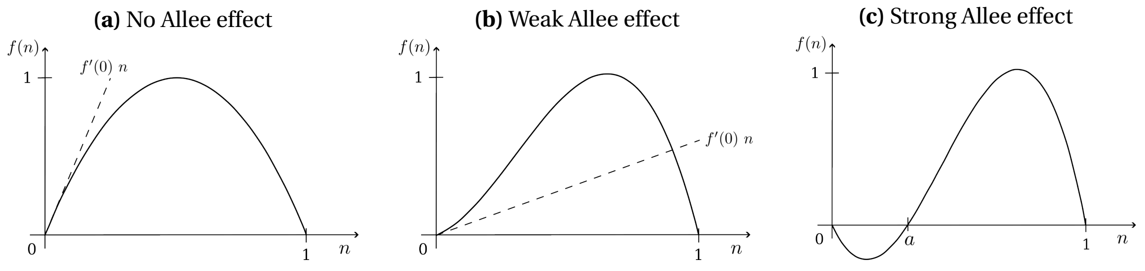

Figure S2: Illustration of the three cases concerning the Allee effect.

C Models

C.1 Growth term details

Parents	Gametes	Adult	Fitness	Growth term
WW + WW	1 → W,W + W,W — 1 →	WW	1	$\frac{n_{WW} n_{WW}}{n}$
WW + WD	c → W,W + D,D — 1 →	WD	$1-sh$	$c\ (1-sh)\ \frac{2n_{WW} n_{DW}}{n}$
	$1-c$ → W,W + W,D — $\frac{1}{2}$ →	WW	1	$(1-c)\ \frac{1}{2}\ \frac{2n_{WW} n_{DW}}{n}$
	$1-c$ → W,W + W,D — $\frac{1}{2}$ →	WD	$1-sh$	$(1-c)\ \frac{1}{2}\ (1-sh)\ \frac{2n_{WW} n_{DW}}{n}$
WW + DD	1 → W,W + D,D — 1 →	WD	$1-sh$	$(1-sh)\ \frac{2n_{WW} n_{DD}}{n}$
WD + WD	c^2 → D,D + D,D — 1 →	DD	$1-s$	$c^2\ (1-s)\ \frac{n_{DW} n_{DW}}{n}$
	$2c(1-c)$ → D,D + W,D — $\frac{1}{2}$ →	DD	$1-s$	$c\,(1-c)\ (1-s)\ \frac{n_{DW} n_{DW}}{n}$
	$2c(1-c)$ → D,D + W,D — $\frac{1}{2}$ →	WD	$1-sh$	$c\,(1-c)\ (1-sh)\ \frac{n_{DW} n_{DW}}{n}$
	$(1-c)^2$ → W,D + W,D — $\frac{1}{4}$ →	DD	$1-s$	$(1-c)^2\ \frac{1}{4}\ (1-s)\ \frac{n_{DW} n_{DW}}{n}$
	$(1-c)^2$ → W,D + W,D — $\frac{1}{2}$ →	WD	$1-sh$	$(1-c)^2\ \frac{1}{2}\ (1-sh)\ \frac{n_{DW} n_{DW}}{n}$
	$(1-c)^2$ → W,D + W,D — $\frac{1}{4}$ →	WW	1	$(1-c)^2\ \frac{1}{4}\ \frac{n_{DW} n_{DW}}{n}$
WD + DD	c → D,D + D,D — 1 →	DD	$1-s$	$c\ (1-s)\ \frac{2n_{DW} n_{DD}}{n}$
	$1-c$ → W,D + D,D — $\frac{1}{2}$ →	WD	$1-sh$	$(1-c)\ \frac{1}{2}\ (1-sh)\ \frac{2n_{DW} n_{DD}}{n}$
	$1-c$ → W,D + D,D — $\frac{1}{2}$ →	DD	$1-s$	$(1-c)\ \frac{1}{2}\ (1-s)\ \frac{2n_{DW} n_{DD}}{n}$
DD + DD	1 → D,D + D,D — 1 →	DD	$1-s$	$(1-s)\ \frac{n_{DD} n_{DD}}{n}$

Table S1: Growth term details when conversion occurs in the germline.

C.2 Genotype densities

For the sake of clarity, we omit variables in the notation ($n_i = n_i(t,x)$) in the following. Each model contains three equations for the three genotype densities: homozygote drive n_{DD}, heterozygote n_{DW} and homozygote wild-type n_{WW}.

Model BN:

$$\begin{cases} \partial_t n_{DD} = (1-s)\big(r\,(1-n)+1\big)\,\dfrac{\frac{1}{4}\,(1+c)^2\,n_{DW}^2 + (1+c)\,n_{DW}n_{DD} + n_{DD}^2}{n} - n_{DD} + \partial_{xx}^2 n_{DD}, \\[2ex] \partial_t n_{DW} = (1-sh)\big(r\,(1-n)+1\big)\,\dfrac{(1+c)\,n_{WW}n_{DW} + 2\,n_{WW}n_{DD} + \frac{1}{2}\,(1-c^2)\,n_{DW}^2 + (1-c)\,n_{DW}n_{DD}}{n} - n_{DW} + \partial_{xx}^2 n_{DW}, \\[2ex] \partial_t n_{WW} = \big(r\,(1-n)+1\big)\,\dfrac{n_{WW}^2 + (1-c)\,n_{WW}n_{DW} + \frac{1}{4}\,(1-c)^2\,n_{DW}^2}{n} - n_{WW} + \partial_{xx}^2 n_{WW}. \end{cases} \tag{C.1}$$

Model BA:

$$\begin{cases} \partial_t n_{DD} = (1-s)\big(\max(r(1-n)(n-a)+1,0)\big)\,\dfrac{\frac{1}{4}\,(1+c)^2\,n_{DW}^2 + (1+c)\,n_{DW}n_{DD} + n_{DD}^2}{n} - n_{DD} + \partial_{xx}^2 n_{DD}, \\[2ex] \partial_t n_{DW} = (1-sh)\big(\max(r(1-n)(n-a)+1,0)\big)\,\dfrac{(1+c)\,n_{WW}n_{DW} + 2\,n_{WW}n_{DD} + \frac{1}{2}\,(1-c^2)\,n_{DW}^2 + (1-c)\,n_{DW}n_{DD}}{n} - n_{DW} + \partial_{xx}^2 n_{DW}, \\[2ex] \partial_t n_{WW} = \big(\max(r(1-n)(n-a)+1,0)\big)\,\dfrac{n_{WW}^2 + (1-c)\,n_{WW}n_{DW} + \frac{1}{4}\,(1-c)^2\,n_{DW}^2}{n} - n_{WW} + \partial_{xx}^2 n_{WW}. \end{cases} \tag{C.2}$$

Model DN:

$$\begin{cases} \partial_t n_{DD} = (1-s)\big(r+1\big)\,\dfrac{\frac{1}{4}\,(1+c)^2\,n_{DW}^2 + (1+c)\,n_{DW}n_{DD} + n_{DD}^2}{n} - \big(rn+1\big)n_{DD} + \partial_{xx}^2 n_{DD}, \\[2ex] \partial_t n_{DW} = (1-sh)\big(r+1\big)\,\dfrac{(1+c)\,n_{WW}n_{DW} + 2\,n_{WW}n_{DD} + \frac{1}{2}\,(1-c^2)\,n_{DW}^2 + (1-c)\,n_{DW}n_{DD}}{n} - \big(rn+1\big)n_{DW} + \partial_{xx}^2 n_{DW}, \\[2ex] \partial_t n_{WW} = \big(r+1\big)\,\dfrac{n_{WW}^2 + (1-c)\,n_{WW}n_{DW} + \frac{1}{4}\,(1-c)^2\,n_{DW}^2}{n} - \big(rn+1\big)n_{WW} + \partial_{xx}^2 n_{WW}. \end{cases} \tag{C.3}$$

Model DA:

$$\begin{cases} \partial_t n_{DD} = (1-s)\big(r+1\big)\,\dfrac{\frac{1}{4}\,(1+c)^2\,n_{DW}^2 + (1+c)\,n_{DW}n_{DD} + n_{DD}^2}{n} - (r(n-1)(n-a)+r+1)\,n_{DD} + \partial_{xx}^2 n_{DD}, \\[2ex] \partial_t n_{DW} = (1-sh)\big(r+1\big)\,\dfrac{(1+c)\,n_{WW}n_{DW} + 2\,n_{WW}n_{DD} + \frac{1}{2}\,(1-c^2)\,n_{DW}^2 + (1-c)\,n_{DW}n_{DD}}{n} - (r(n-1)(n-a)+r+1)\,n_{DW} + \partial_{xx}^2 n_{DW}, \\[2ex] \partial_t n_{WW} = \big(r+1\big)\,\dfrac{n_{WW}^2 + (1-c)\,n_{WW}n_{DW} + \frac{1}{4}\,(1-c)^2\,n_{DW}^2}{n} - (r(n-1)(n-a)+r+1)\,n_{WW} + \partial_{xx}^2 n_{WW}. \end{cases} \tag{C.4}$$

Note that all four models reduce to a single model for $r = 0$. This model is:

$$\begin{cases} \partial_t n_{DD} = (1-s)\,\dfrac{\frac{1}{4}\,(1+c)^2\,n_{DW}^2 + (1+c)\,n_{DW}n_{DD} + n_{DD}^2}{n} - n_{DD} + \partial_{xx}^2 n_{DD}, \\[2ex] \partial_t n_{DW} = (1-sh)\,\dfrac{(1+c)\,n_{WW}n_{DW} + 2\,n_{WW}n_{DD} + \frac{1}{2}\,(1-c^2)\,n_{DW}^2 + (1-c)\,n_{DW}n_{DD}}{n} - n_{DW} + \partial_{xx}^2 n_{DW}, \\[2ex] \partial_t n_{WW} = \dfrac{n_{WW}^2 + (1-c)\,n_{WW}n_{DW} + \frac{1}{4}\,(1-c)^2\,n_{DW}^2}{n} - n_{WW} + \partial_{xx}^2 n_{WW}. \end{cases} \tag{C.5}$$

C.3 Allelic densities

For our analysis, it is convenient to introduce the allelic (half-) densities (n_D, n_W). For a conversion occurring in the germline, we have $n_D = n_{DD} + \frac{1+c}{2}\,n_{DW}$ and $n_W = n_{WW} + (1 - \frac{1+c}{2})\,n_{DW}$ (see section 3.2 in [38] for more details). We deduce the following systems.

Model BN:

$$\begin{cases} \partial_t n_D = n_D\Big[\dfrac{r\,(1-n)+1}{n}\Big[(1-s)n_D + (1-sh)\,(1+c)\,n_W\Big] - 1\Big] + \partial_{xx}^2 n_D, \\[2ex] \partial_t n_W = n_W\Big[\dfrac{r\,(1-n)+1}{n}\Big[n_W + (1-sh)\,(1-c)\,n_D\Big] - 1\Big] + \partial_{xx}^2 n_W. \end{cases} \tag{C.6}$$

Model BA:

$$\begin{cases} \partial_t n_D = n_D\Big[\dfrac{\max(r(1-n)(n-a)+1,0)}{n}\Big[(1-s)n_D + (1-sh)\,(1+c)\,n_W\Big] - 1\Big] + \partial_{xx}^2 n_D, \\[2ex] \partial_t n_W = n_W\Big[\dfrac{\max(r(1-n)(n-a)+1,0)}{n}\Big[n_W + (1-sh)\,(1-c)\,n_D\Big] - 1\Big] + \partial_{xx}^2 n_W. \end{cases} \tag{C.7}$$

Model DN:

$$\begin{cases} \partial_t n_{\mathrm{D}} = n_{\mathrm{D}} \left[\frac{r+1}{n}\left[(1-s)n_{\mathrm{D}} + (1-sh)(1+c)\, n_{\mathrm{W}}\right] - \left(rn+1\right)\right] + \partial^2_{xx} n_{\mathrm{D}}, \\ \partial_t n_{\mathrm{W}} = n_{\mathrm{W}} \left[\frac{r+1}{n}\left[n_{\mathrm{W}} + (1-sh)(1-c)\, n_{\mathrm{D}}\right] - \left(rn+1\right)\right] + \partial^2_{xx} n_{\mathrm{W}}. \end{cases} \tag{C.8}$$

Model DA:

$$\begin{cases} \partial_t n_{\mathrm{D}} = n_{\mathrm{D}} \left[\frac{r+1}{n}\left[(1-s)n_{\mathrm{D}} + (1-sh)(1+c)\, n_{\mathrm{W}}\right] - (r(n-1)(n-a)+r+1)\right] + \partial^2_{xx} n_{\mathrm{D}}, \\ \partial_t n_{\mathrm{W}} = n_{\mathrm{W}} \left[\frac{r+1}{n}\left[n_{\mathrm{W}} + (1-sh)(1-c)\, n_{\mathrm{D}}\right] - (r(n-1)(n-a)+r+1)\right] + \partial^2_{xx} n_{\mathrm{W}}. \end{cases} \tag{C.9}$$

C.4 Allelic frequencies

It may sometimes be more appropriate to write the models in terms of the proportion of drive allele $p_{\mathrm{D}} = \frac{n_{\mathrm{D}}}{n_{\mathrm{D}}+n_{\mathrm{W}}}$ and total population size $n = n_{\mathrm{D}} + n_{\mathrm{W}}$. The models become the following.

Model BN:

$$\begin{cases} \partial_t n = \left(r\,(1-n)+1\right)\left((1-s)\, p_{\mathrm{D}}^2 + 2\,(1-sh)\, p_{\mathrm{D}}\,(1-p_{\mathrm{D}}) + (1-p_{\mathrm{D}})^2\right) n - n + \partial^2_{xx} n, \\ \partial_t p_{\mathrm{D}} = \left(r\,(1-n)+1\right)\left((2h-1)\, s\, p_{\mathrm{D}} + (1-sh)(1+c) - 1\right) p_{\mathrm{D}}\,(1-p_{\mathrm{D}}) + 2\,\partial_x \log(n)\,\partial_x p_{\mathrm{D}} + \partial^2_{xx} p_{\mathrm{D}}. \end{cases} \tag{C.10}$$

Model BA:

$$\begin{cases} \partial_t n = \left(\max(r(1-n)(n-a)+1,0)\right)\left((1-s)\, p_{\mathrm{D}}^2 + 2\,(1-sh)\, p_{\mathrm{D}}\,(1-p_{\mathrm{D}}) + (1-p_{\mathrm{D}})^2\right) n - n + \partial^2_{xx} n, \\ \partial_t p_{\mathrm{D}} = \left(\max(r(1-n)(n-a)+1,0)\right)\left((2h-1)\, s\, p_{\mathrm{D}} + (1-sh)(1+c) - 1\right) p_{\mathrm{D}}\,(1-p_{\mathrm{D}}) + 2\,\partial_x \log(n)\,\partial_x p_{\mathrm{D}} + \partial^2_{xx} p_{\mathrm{D}}. \end{cases} \tag{C.11}$$

Model DN:

$$\begin{cases} \partial_t n = \left(r+1\right)\left((1-s)\, p_{\mathrm{D}}^2 + 2\,(1-sh)\, p_{\mathrm{D}}\,(1-p_{\mathrm{D}}) + (1-p_{\mathrm{D}})^2\right) n - \left(rn+1\right) n + \partial^2_{xx} n, \\ \partial_t p_{\mathrm{D}} = \left(r+1\right)\left((2h-1)\, s\, p_{\mathrm{D}} + (1-sh)(1+c) - 1\right) p_{\mathrm{D}}\,(1-p_{\mathrm{D}}) + 2\,\partial_x \log(n)\,\partial_x p_{\mathrm{D}} + \partial^2_{xx} p_{\mathrm{D}}. \end{cases} \tag{C.12}$$

Model DA:

$$\begin{cases} \partial_t n = \left(r+1\right)\left((1-s)\, p_{\mathrm{D}}^2 + 2\,(1-sh)\, p_{\mathrm{D}}\,(1-p_{\mathrm{D}}) + (1-p_{\mathrm{D}})^2\right) n - (r(n-1)(n-a)+r+1)\, n + \partial^2_{xx} n, \\ \partial_t p_{\mathrm{D}} = \left(r+1\right)\left((2h-1)\, s\, p_{\mathrm{D}} + (1-sh)(1+c) - 1\right) p_{\mathrm{D}}\,(1-p_{\mathrm{D}}) + 2\,\partial_x \log(n)\,\partial_x p_{\mathrm{D}} + \partial^2_{xx} p_{\mathrm{D}}. \end{cases} \tag{C.13}$$

Equations on p_{D} differ from the standard equation often used in populations genetics, as they contain an advection term $2\,\partial_x(\log n)\,\partial_x p_{\mathrm{D}}$. This term appears when calculating $\partial^2_{xx} p_{\mathrm{D}} = \partial^2_{xx} \frac{n_{\mathrm{DD}}}{n}$ and represents a demographic flux from denser to less dense areas, due to variations in population density. It is opposed to the spread of the costly drive allele (see Figure 2 [11]).

C.5 Final allelic proportions for r small and large

In models BN and BA for large values of r, using the Strugarek-Vauchelet rescaling [84] in (C.10) and (C.11), the systems reduce to one limit equation on p_{D}:

$$\partial_t p_{\mathrm{D}} = \frac{\left((2h-1)\, s\, p_{\mathrm{D}} + (1-sh)(1+c) - 1\right) p_{\mathrm{D}}\,(1-p_{\mathrm{D}})}{(1-s)\, p_{\mathrm{D}}^2 + 2\,(1-sh)\, p_{\mathrm{D}}\,(1-p_{\mathrm{D}}) + (1-p_{\mathrm{D}})^2} + \partial^2_{xx} p_{\mathrm{D}}. \tag{C.14}$$

Equation (C.14) has been previously introduced and studied in [22] and [18], but without spatial structure (panmictic model). Notably, Figure 4 in [18] presents a heatmap illustrating the final proportions for the case $c = 0.85$. Figure S3 summarises the connections between this panmictic model, its polynomial approximation under weak selection and Models BN and BA.

Models BN and BA without space

$$\begin{cases} \partial_t n = B(n)\Big((1-s)\,p_{\mathrm{D}}^2 + 2\,(1-sh)\,p_{\mathrm{D}}\,(1-p_{\mathrm{D}}) + (1-p_{\mathrm{D}})^2\Big)n - D(n)n, \\ \partial_t p_{\mathrm{D}} = B(n)\Big((2h-1)\,s\,p_{\mathrm{D}} + (1-sh)(1+c) - 1\Big)\,p_{\mathrm{D}}\,(1-p_{\mathrm{D}}). \end{cases}$$

↓ when $r \to +\infty$

Panmictic model introduced by Deredec et al. [22]

$$\begin{cases} n = 1, \\ \partial_t p_{\mathrm{D}} = \dfrac{(1-s)p_{\mathrm{D}}^2 + (1-sh)(1+c)p_{\mathrm{D}}(1-p_{\mathrm{D}})}{(1-s)\,p_{\mathrm{D}}^2 + 2\,(1-sh)\,p_{\mathrm{D}}\,(1-p_{\mathrm{D}}) + (1-p_{\mathrm{D}})^2} - p_{\mathrm{D}} = \dfrac{\Big((2h-1)\,s\,p_{\mathrm{D}} + (1-sh)(1+c) - 1\Big)\,p_{\mathrm{D}}\,(1-p_{\mathrm{D}})}{(1-s)\,p_{\mathrm{D}}^2 + 2\,(1-sh)\,p_{\mathrm{D}}\,(1-p_{\mathrm{D}}) + (1-p_{\mathrm{D}})^2}. \end{cases}$$

↓ under the assumption of weak selection ($s \simeq 0$)

Polynomial approximation

$$\begin{cases} n = 1, \\ \partial_t p_{\mathrm{D}} = \Big((2h-1)\,s\,p_{\mathrm{D}} + (1-sh)(1+c) - 1\Big)\,p_{\mathrm{D}}\,(1-p_{\mathrm{D}}). \end{cases}$$

Figure S3: Relationships between Models BN and BA, a panmictic model introduced in [22] and its polynomial approximation under weak selection.

In models DN and DA, the equation on p_{D} in (C.10) and (C.11) is:

$$\partial_t p_{\mathrm{D}} = \underbrace{(r+1)\Big((2h-1)\,s\,p_{\mathrm{D}} + (1-sh)(1+c) - 1\Big)p_{\mathrm{D}}\,(1-p_{\mathrm{D}})}_{\text{reaction term}} + 2\,\partial_x \log(n)\,\partial_x p_{\mathrm{D}} + \partial_{xx}^2 p_{\mathrm{D}}. \tag{C.15}$$

The reaction term in equation (C.15) becomes larger as r increases: this indicates that the traveling wave has an infinite speed when r tends to infinity, meaning that the equilibrium is reached instantaneously. Therefore, the term $2\,\partial_x \log(n)\,\partial_x p_{\mathrm{D}}$ is instantaneously zero and the final proportions are the same as for models BN and BA.

As a consequence, all models BN, BA, DN and DA share the same final proportions for large values of r. This conclusion also holds for $r = 0$, as the models are equal (see Appendix C.2). Final allelic proportions for $r = 0$ and large values of r have already been determined in a previous article [38], for the BN case. We recall these results in section 3.1 and generalise them to our four models.

C.6 Speed of the problem simplified at low drive density

In Section 3.3, we focus on drive invasion and therefore consider low drive density and high wild-type density at the front of the wave. The speed v of the wave can be calculated when the models are simplified (linearised) at low drive density: it is deduced from the reproduction of the few drive individuals at the front of the wave,

$$v = 2\sqrt{\lim_{n_{\mathrm{D}} \to 0} \left(\frac{F(n_{\mathrm{D}})}{n_{\mathrm{D}}}\right)}, \tag{C.16}$$

where F represents the net production of drive alleles. The formulas for F in the different models are the following.

Model BN:

$$F^{(\mathrm{BN})}(n_{\mathrm{D}}) = n_{\mathrm{D}}\Big[(r\,(1-n)+1)\Big[(1-s)\frac{n_{\mathrm{D}}}{n} + (1-sh)\,(1+c)\,\frac{n_{\mathrm{W}}}{n}\Big] - 1\Big] \tag{C.17}$$

Model BA:

$$F^{(\mathrm{BA})}(n_{\mathrm{D}}) = n_{\mathrm{D}}\left[\max(r(1-n)(n-a)+1,0)\left[(1-s)\frac{n_{\mathrm{D}}}{n}+(1-sh)(1+c)\frac{n_{\mathrm{W}}}{n}\right]-1\right]. \tag{C.18}$$

Model DN:

$$F^{(\mathrm{DN})}(n_{\mathrm{D}}) = n_{\mathrm{D}}\left[(r+1)\left[(1-s)\frac{n_{\mathrm{D}}}{n}+(1-sh)(1+c)\frac{n_{\mathrm{W}}}{n}\right]-(rn+1)\right]. \tag{C.19}$$

Model DA:

$$F^{(\mathrm{DA})}(n_{\mathrm{D}}) = n_{\mathrm{D}}\left[(r+1)\left[(1-s)\frac{n_{\mathrm{D}}}{n}+(1-sh)(1+c)\frac{n_{\mathrm{W}}}{n}\right]-(r(n-1)(n-a)+r+1)\right]. \tag{C.20}$$

Considering high wild-type density at the front of the wave ($n_{\mathrm{W}} \approx n \approx 1$), the speed in models BN and BA is given by:

$$v^{(\mathrm{B})} = 2\sqrt{(1-sh)(1+c)-1}. \tag{C.21}$$

In models DN and DA, we have:

$$v^{(\mathrm{D})} = 2\sqrt{(1+r)\left[(1-sh)(1+c)-1\right]}. \tag{C.22}$$

To understand why $v^{(\mathrm{D})}$ is greater by a coefficient of $\sqrt{1+r}$ than $v^{(\mathrm{B})}$, we have to understand the population dynamics at the front of the wave. There, the density is close to the maximum carrying capacity 1, with low drive density ($n_{\mathrm{D}} \approx 0$) and high wild-type density ($n_{\mathrm{W}} \approx n \approx 1$). On one hand, in models BN and BA, the density-dependence constraint is placed on the birth term, reducing the production rate of drive alleles to $(1-sh)$ $(1+c)$ while individuals disappear at rate 1 (C.17, C.18). On the other hand, in models DN and DA, the density-dependence constraint is placed on the death term increasing to $(r+1)$ the rate at which the drive alleles disappears, while they are produced at rate $(r+1)(1-sh)(1+c)$ (C.19, C.20). Consequently, the net production remains constant $(1-sh)(1+c)$, but the turnover rate is $r+1$ times greater. As the wave movement largely relies on the reproduction, this reflects in the speed formula: the propagation is $\sqrt{r+1}$ times faster.

D Comparison of the conditions leading to eradication

To compare the conditions leading to eradication, we refer to the results summarised in Table 3.

In Models BN and DN, eradication occurs when:

$$r\mathscr{F}(p_{\mathrm{D}}^{*}) < 1-\mathscr{F}(p_{\mathrm{D}}^{*}), \tag{D.1}$$

in Model BA, when:

$$\left(\frac{1-a}{2}\right)^{2} r\mathscr{F}(p_{\mathrm{D}}^{*}) < 1-\mathscr{F}(p_{\mathrm{D}}^{*}). \tag{D.2}$$

and in Model DA, when:

$$r\mathscr{F}(p_{\mathrm{D}}^{*}) - r\left[1-\left(\frac{1-a}{2}\right)^{2}\right] < 1-\mathscr{F}(p_{\mathrm{D}}^{*}), \tag{D.3}$$

With $\left(\frac{1-a}{2}\right)^{2} \in [0,1]$ and $\mathscr{F}(p_{\mathrm{D}}^{*}) \in [1-s,1]$, we obtain the following inequalities:

$$r\mathscr{F}(p_{\mathrm{D}}^{*}) - r\left[1-\left(\frac{1-a}{2}\right)^{2}\right] \le \left(\frac{1-a}{2}\right)^{2} r\mathscr{F}(p_{\mathrm{D}}^{*}) \le r\mathscr{F}(p_{\mathrm{D}}^{*}) \tag{D.4}$$

Inequalities (D.2) and (D.3) both imply (D.1). In other words, there is a greater range of parameters leading to eradication in Models BA and DA (with Allee effect) than in Models BN and DN (without Allee effect).

Inequality (D.3) also implies (D.2), i.e., in models with Allee effect, for a given a value, there is a greater range of parameters leading to eradication when the density-dependence constraint acts on the deaths (Model DA) than when it acts on the births (Model BA).

Note that when $a = -1$, the conditions leading to eradication are equivalent in all four models . When a becomes larger, the range of parameters leading to eradication in models BA and DA also becomes wider.

E Comparison of the conditions leading to persistence

To compare the conditions leading to persistence, we refer to the results summarised in Table 3.

In Models BN and DN, persistence occurs when:

$$r\mathscr{F}(p^*_{\text{D}}) > 1-\mathscr{F}(p^*_{\text{D}}), \tag{E.1}$$

in Model BA, when:

$$\begin{cases} \left(\frac{1-a}{2}\right)^2 r\mathscr{F}(p^*_{\text{D}}) > 1-\mathscr{F}(p^*_{\text{D}}), \\ -a\ r\ \mathscr{F}(p^*_{\text{D}}) > 1-\mathscr{F}(p^*_{\text{D}}), \end{cases} \tag{E.2}$$

and in Model DA, when:

$$\begin{cases} r\mathscr{F}(p^*_{\text{D}}) - r\left[1-\left(\frac{1-a}{2}\right)^2\right] > 1-\mathscr{F}(p^*_{\text{D}}), \\ r\mathscr{F}(p^*_{\text{D}}) - r(a+1) > 1-\mathscr{F}(p^*_{\text{D}}). \end{cases} \tag{E.3}$$

First note that persistence is only possible in case of a weak Allee effect ($a < 0$) in Models BA and DA (second lines in Systems (E.2) and (E.3)). With $\left(\frac{1-a}{2}\right)^2 \in [0,1]$ and $\mathscr{F}(p^*_{\text{D}}) \in [1-s,1]$, we obtain the following inequalities:

$$r\mathscr{F}(p^*_{\text{D}}) \geq \left(\frac{1-a}{2}\right)^2 r\mathscr{F}(p^*_{\text{D}}) \geq r\mathscr{F}(p^*_{\text{D}}) - r\left[1-\left(\frac{1-a}{2}\right)^2\right] \tag{E.4}$$

$$r\mathscr{F}(p^*_{\text{D}}) \geq -a\,r\mathscr{F}(p^*_{\text{D}}) \geq r\mathscr{F}(p^*_{\text{D}}) - r(1+a) \tag{E.5}$$

Inequality (E.1) imply both (E.2) and (E.3). In other words, there is a greater range of parameters leading to persistence in Models BN and DN (without Allee effect) than in Models BA and DA (with Allee effect).

Inequality (E.2) also implies (E.3), i.e., in models with Allee effect, for a given a value, there is a greater range of parameters leading to eradication when the density-dependence constraint acts on the births (Model BA) than when it acts on the deaths (Model DA).

Note that when $a = -1$, the conditions leading to persistence are equivalent in all four models. When a becomes larger, the range of parameters leading to persistence in models BA and DA becomes smaller. In case of a strong Allee effect ($a > 0$), the "persistence" regime disappears.

F Comparison of the final densities in case of persistence

F.1 Comparison of the final density in models BN and DN

We compare the final densities for models BN and DN given in Table 3 in case of persistence.

$$n^{*(\text{DN})} = 1 - \frac{(r+1)(1-\mathscr{F}(p^*_{\text{D}}))}{r} = 1 - \frac{-r\mathscr{F}(p^*_{\text{D}}) + r + 1 - \mathscr{F}(p^*_{\text{D}})}{r} = \mathscr{F}(p^*_{\text{D}}) - \frac{1-\mathscr{F}(p^*_{\text{D}})}{r} = \mathscr{F}(p^*_{\text{D}})\, n^{*(\text{BN})} \tag{F.1}$$

with $\mathscr{F}(p^*_{\text{D}}) \in [1-s,1]$. The final density is $\mathscr{F}(p^*_{\text{D}})$ times lower in model DN than in model BN, in case of persistence. Note that $\mathscr{F}(p^*_{\text{D}}) = 1-s$ in case of a drive invasion, and $\mathscr{F}(p^*_{\text{D}}) \in (1-s,1)$ in case of coexistence.

F.2 Comparison of the final density in models BA and DA

We compare the final densities for model BA and DA in case of persistence given in Table 3.

$$n^{*(\text{BA})} = \frac{1+a+\sqrt{(1+a)^2 - 4(a+1-n^{*(\text{BN})})}}{2} \quad \text{and} \quad n^{*(\text{DA})} = \frac{1+a+\sqrt{(1+a)^2 - 4(a+1-n^{*(\text{DN})})}}{2} \tag{F.2}$$

From the previous section F.1, we know that $n^{*(\text{DN})} = \mathscr{F}(p^*_{\text{D}})\, n^{*(\text{BN})}$ with $\mathscr{F}(p^*_{\text{D}}) \in [1-s,1]$. Therefore:

$$n^{*(\text{DA})} \leq n^{*(\text{BA})} \tag{F.3}$$

This inequality is strict in case of a drive invasion $\mathscr{F}(p^*_{\text{D}}) = 1-s$ or in case of coexistence $\mathscr{F}(p^*_{\text{D}}) \in (1-s,1)$. We derive both density as functions of a, in case of drive persistence $\mathscr{F}(p^*_{\text{D}}) < 1$:

$$\partial_a n^{*(\mathsf{BA})} = 1 - \frac{2(1-a)}{2\sqrt{(1-a)^2 - 4(1-n^{*(\mathsf{BN})})}} < 0 \quad \text{and} \quad \partial_a n^{*(\mathsf{DA})} = 1 - \frac{2(1-a)}{2\sqrt{(1-a)^2 - 4(1-n^{*(\mathsf{DN})})}} < 0, \quad \text{(F.4)}$$

as $n^{*(\mathsf{BN})} < 1$ and $n^{*(\mathsf{DN})} < 1$ when $\mathscr{F}(p^*_{\text{D}}) < 1$ (see Table 3). In other words in case of a drive persistence, the stronger the Allee effect, the smaller the final population density in models BA and DA.

References

[1] Luke S. Alphey et al. "Standardizing the Definition of Gene Drive". In: *Proceedings of the National Academy of Sciences* 117.49 (Dec. 2020), pp. 30864–30867. DOI: 10.1073/pnas.2020417117.

[2] Austin Burt. "Site-Specific Selfish Genes as Tools for the Control and Genetic Engineering of Natural Populations." In: *Proceedings of the Royal Society B: Biological Sciences* 270.1518 (May 2003), pp. 921–928. ISSN: 0962-8452. DOI: 10.1098/rspb.2002.2319.

[3] Austin Burt and Andrea Crisanti. "Gene Drive: Evolved and Synthetic". In: *ACS Chemical Biology* 13.2 (Feb. 2018), pp. 343–346. ISSN: 1554-8929. DOI: 10.1021/acschembio.7b01031.

[4] James E. DiCarlo et al. "Safeguarding CRISPR-Cas9 Gene Drives in Yeast". In: *Nature Biotechnology* 33.12 (Dec. 2015), pp. 1250–1255. ISSN: 1546-1696. DOI: 10.1038/nbt.3412.

[5] Silke Fuchs et al. "Resistance to a CRISPR-based Gene Drive at an Evolutionarily Conserved Site Is Revealed by Mimicking Genotype Fixation". In: *PLOS Genetics* 17.10 (Oct. 2021), e1009740. ISSN: 1553-7404. DOI: 10.1371/journal.pgen.1009740.

[6] Emily Yang et al. "A Homing Suppression Gene Drive with Multiplexed gRNAs Maintains High Drive Conversion Efficiency and Avoids Functional Resistance Alleles". In: *G3 Genes|Genomes|Genetics* 12.6 (June 2022), jkac081. ISSN: 2160-1836. DOI: 10.1093/g3journal/jkac081.

[7] Jackson Champer et al. "Novel CRISPR/Cas9 Gene Drive Constructs Reveal Insights into Mechanisms of Resistance Allele Formation and Drive Efficiency in Genetically Diverse Populations". In: *PLOS Genetics* 13.7 (July 2017), e1006796. ISSN: 1553-7404. DOI: 10.1371/journal.pgen.1006796.

[8] Kyros Kyrou et al. "A CRISPR–Cas9 Gene Drive Targeting Doublesex Causes Complete Population Suppression in Caged Anopheles Gambiae Mosquitoes". In: *Nature Biotechnology* 36.11 (Nov. 2018), pp. 1062–1066. ISSN: 1546-1696. DOI: 10.1038/nbt.4245.

[9] Andrew Hammond et al. "Gene-Drive Suppression of Mosquito Populations in Large Cages as a Bridge between Lab and Field". In: *Nature Communications* 12.1 (July 2021), p. 4589. ISSN: 2041-1723. DOI: 10.1038/s41467-021-24790-6.

[10] H. Charles J. Godfray, Ace North, and Austin Burt. "How Driving Endonuclease Genes Can Be Used to Combat Pests and Disease Vectors". In: *BMC Biology* 15.1 (Sept. 2017), p. 81. ISSN: 1741-7007. DOI: 10.1186/s12915-017-0420-4.

[11] Léo Girardin and Florence Débarre. "Demographic Feedbacks Can Hamper the Spatial Spread of a Gene Drive". In: *Journal of Mathematical Biology* 83.6 (Dec. 2021), p. 67. ISSN: 1432-1416. DOI: 10.1007/s00285-021-01702-2.

[12] Ethan Bier. "Gene Drives Gaining Speed". In: *Nature Reviews Genetics* 23.1 (Jan. 2022), pp. 5–22. ISSN: 1471-0064. DOI: 10.1038/s41576-021-00386-0.

[13] Bruce A. Hay, Georg Oberhofer, and Ming Guo. "Engineering the Composition and Fate of Wild Populations with Gene Drive". In: *Annual Review of Entomology* 66.1 (2021), pp. 407–434. DOI: 10.1146/annurev-ento-020117-043154.

[14] Tony Nolan. "Control of Malaria-Transmitting Mosquitoes Using Gene Drives". In: *Philosophical Transactions of the Royal Society B: Biological Sciences* 376.1818 (Dec. 2020), p. 20190803. DOI: 10.1098/rstb.2019.0803.

[15] Valentino Gantz et al. "Highly Efficient Cas9-mediated Gene Drive for Population Modification of the Malaria Vector Mosquito Anopheles Stephensi". In: *Proceedings of the National Academy of Sciences* 112 (Nov. 2015). DOI: 10.1073/pnas.1521077112.

[16] Andrew Hammond et al. "A CRISPR-Cas9 Gene Drive System Targeting Female Reproduction in the Malaria Mosquito Vector Anopheles Gambiae". In: *Nature biotechnology* 34 (Dec. 2015). DOI: 10.1038/nbt.3439.

[17] Kevin M Esvelt et al. "Concerning RNA-guided Gene Drives for the Alteration of Wild Populations". In: *eLife* 3 (July 2014). Ed. by Diethard Tautz, e03401. ISSN: 2050-084X. DOI: 10.7554/eLife.03401.

[18] Nicolas O. Rode et al. "Population Management Using Gene Drive: Molecular Design, Models of Spread Dynamics and Assessment of Ecological Risks". In: *Conservation Genetics* 20.4 (Aug. 2019), pp. 671–690. ISSN: 1572-9737. DOI: 10.1007/s10592-019-01165-5.

[19] Bhagyashree Kaduskar et al. “Reversing Insecticide Resistance with Allelic-Drive in Drosophila Melanogaster”. In: *Nature Communications* 13.1 (Jan. 2022), p. 291. ISSN: 2041-1723. DOI: 10.1038/s41467-021-27654-1.

[20] Paul Neve. “Gene Drive Systems: Do They Have a Place in Agricultural Weed Management?” In: *Pest Management Science* 74.12 (2018), pp. 2671–2679. ISSN: 1526-4998. DOI: 10.1002/ps.5137.

[21] Sumit Dhole, Alun L. Lloyd, and Fred Gould. “Gene Drive Dynamics in Natural Populations: The Importance of Density Dependence, Space, and Sex”. In: *Annual Review of Ecology, Evolution, and Systematics* 51.1 (Nov. 2020), pp. 505–531. ISSN: 1543-592X. DOI: 10.1146/annurev-ecolsys-031120-101013.

[22] Anne Deredec, Austin Burt, and H. C. J. Godfray. “The Population Genetics of Using Homing Endonuclease Genes in Vector and Pest Management”. In: *Genetics* 179.4 (Aug. 2008), pp. 2013–2026. ISSN: 0016-6731. DOI: 10.1534/genetics.108.089037.

[23] Robert L Unckless et al. “Modeling the Manipulation of Natural Populations by the Mutagenic Chain Reaction”. In: *Genetics* 201.2 (Oct. 2015), pp. 425–431. ISSN: 1943-2631. DOI: 10.1534/genetics.115.177592.

[24] Tom J. de Jong. “Gene Drives Do Not Always Increase in Frequency: From Genetic Models to Risk Assessment”. In: *Journal of Consumer Protection and Food Safety* 12.4 (Dec. 2017), pp. 299–307. ISSN: 1661-5867. DOI: 10.1007/s00003-017-1131-z.

[25] Hidenori Tanaka, Howard A. Stone, and David R. Nelson. “Spatial Gene Drives and Pushed Genetic Waves”. In: *Proceedings of the National Academy of Sciences of the United States of America* 114.32 (Aug. 2017), pp. 8452–8457. ISSN: 1091-6490. DOI: 10.1073/pnas.1705868114.

[26] R. A. Fisher. “The Wave of Advance of Advantageous Genes”. In: *Annals of Eugenics* 7.4 (1937), pp. 355–369. ISSN: 2050-1439. DOI: 10.1111/j.1469-1809.1937.tb02153.x.

[27] A.N. Kolmogorov, I.G Petrovsky, and N.S. Piskunov. “Étude de l’équation de La Diffusion Avec Croissance de La Quantité de Matière et Son Application à Un Problème Biologigue”. In: *Moscow Univ. Bull. Ser. Internat. Sect. A* 1 (1937), p. 1.

[28] AD Bazykin. “Hypothetical mechanism of speciaton”. In: *Evolution* (1969), pp. 685–687.

[29] N. H. Barton. “The Dynamics of Hybrid Zones”. In: *Heredity* 43.3 (Dec. 1979), pp. 341–359. ISSN: 1365-2540. DOI: 10.1038/hdy.1979.87.

[30] Nicholas H Barton and Godfrey M Hewitt. “Analysis of hybrid zones”. In: *Annual review of Ecology and Systematics* (1985), pp. 113–148.

[31] N. H. Barton and Michael Turelli. “Spatial Waves of Advance with Bistable Dynamics: Cytoplasmic and Genetic Analogues of Allee Effects.” In: *The American Naturalist* 178.3 (Sept. 2011), E48–E75. ISSN: 0003-0147. DOI: 10.1086/661246.

[32] James Mallet et al. “Estimates of selection and gene flow from measures of cline width and linkage disequilibrium in Heliconius hybrid zones.” In: *Genetics* 124.4 (1990), pp. 921–936.

[33] Nicholas H Barton. “The effects of linkage and density-dependent regulation on gene flow”. In: *Heredity* 57.3 (1986), pp. 415–426.

[34] Philip T. Leftwich et al. “Recent Advances in Threshold-Dependent Gene Drives for Mosquitoes”. In: *Biochemical Society Transactions* 46.5 (Sept. 2018), pp. 1203–1212. ISSN: 0300-5127. DOI: 10.1042/BST20180076.

[35] Jackson Champer, Anna Buchman, and Omar S. Akbari. “Cheating Evolution: Engineering Gene Drives to Manipulate the Fate of Wild Populations”. In: *Nature Reviews Genetics* 17.3 (Mar. 2016), pp. 146–159. ISSN: 1471-0064. DOI: 10.1038/nrg.2015.34.

[36] Andrea Beaghton, Pantelis John Beaghton, and Austin Burt. “Gene Drive through a Landscape: Reaction–Diffusion Models of Population Suppression and Elimination by a Sex Ratio Distorter”. In: *Theoretical Population Biology* 108 (Apr. 2016), pp. 51–69. ISSN: 0040-5809. DOI: 10.1016/j.tpb.2015.11.005.

[37] Léo Girardin. “The Effect of Random Dispersal on Competitive Exclusion – A Review”. In: *Mathematical Biosciences* 318 (Dec. 2019), p. 108271. ISSN: 0025-5564. DOI: 10.1016/j.mbs.2019.108271.

[38] Léna Kläy et al. "Pulled, Pushed or Failed: The Demographic Impact of a Gene Drive Can Change the Nature of Its Spatial Spread". In: *Journal of Mathematical Biology* 87.2 (July 2023), p. 30. ISSN: 1432-1416. DOI: 10.1007/s00285-023-01926-4.

[39] Ace R. North, Austin Burt, and H. Charles J. Godfray. "Modelling the Potential of Genetic Control of Malaria Mosquitoes at National Scale". In: *BMC Biology* 17.1 (Mar. 2019), p. 26. ISSN: 1741-7007. DOI: 10.1186/s12915-019-0645-5.

[40] Ace R. North, Austin Burt, and H. Charles J. Godfray. "Modelling the Suppression of a Malaria Vector Using a CRISPR-Cas9 Gene Drive to Reduce Female Fertility". In: *BMC Biology* 18 (Aug. 2020), p. 98. ISSN: 1741-7007. DOI: 10.1186/s12915-020-00834-z.

[41] Richard Levins. "The strategy of model building in population biology". In: *American scientist* 54.4 (1966), pp. 421–431.

[42] Jackson Champer et al. "Population Dynamics of Underdominance Gene Drive Systems in Continuous Space". In: *ACS Synthetic Biology* 9.4 (Apr. 2020), pp. 779–792. DOI: 10.1021/acssynbio.9b00452.

[43] Jackson Champer et al. "Suppression Gene Drive in Continuous Space Can Result in Unstable Persistence of Both Drive and Wild-Type Alleles". In: *Molecular Ecology* 30.4 (Feb. 2021), pp. 1086–1101. ISSN: 1365-294X. DOI: 10.1111/mec.15788.

[44] Mingzuyu Pan and Jackson Champer. *Making Waves: Comparative Analysis of Gene Drive Spread Characteristics in a Continuous Space Model.* Nov. 2022. DOI: 10.1101/2022.11.01.514650.

[45] Jaehee Kim et al. "Incorporating ecology into gene drive modelling". In: *Ecology Letters* 26 (2023), S62–S80.

[46] Claus Rueffler, Martijn Egas, and Johan A. J. Metz. "Evolutionary Predictions Should Be Based on Individual-Level Traits." In: *The American Naturalist* 168.5 (Nov. 2006), E148–E162. ISSN: 0003-0147. DOI: 10.1086/508618.

[47] A. Tsoularis and J. Wallace. "Analysis of Logistic Growth Models". In: *Mathematical Biosciences* 179.1 (July 2002), pp. 21–55. ISSN: 0025-5564. DOI: 10.1016/S0025-5564(02)00096-2.

[48] Nicolas O Rode, Virginie Courtier-Orgogozo, and Florence Débarre. "Can a Population Targeted by a CRISPR-Based Homing Gene Drive Be Rescued?" In: *G3 Genes|Genomes|Genetics* 10.9 (Sept. 2020), pp. 3403–3415. ISSN: 2160-1836. DOI: 10.1534/g3.120.401484.

[49] Gloria M. Luque et al. "The Genetic Allee Effect: A Unified Framework for the Genetics and Demography of Small Populations". In: *Ecosphere* 7.7 (2016), e01413. ISSN: 2150-8925. DOI: 10.1002/ecs2.1413.

[50] Franck Courchamp, Ludek Berec, and Joanna Gascoigne. *Allee Effects in Ecology and Conservation.* OUP Oxford, Feb. 2008. ISBN: 978-0-19-152466-0.

[51] Peter Armbruster, Robert A. Hutchinson, and Trevor Linvell. "Equivalent Inbreeding Depression under Laboratory and Field Conditions in a Tree-Hole-Breeding Mosquito". In: *Proceedings of the Royal Society of London. Series B: Biological Sciences* 267.1456 (Oct. 2000), pp. 1939–1945. DOI: 10.1098/rspb.2000.1233.

[52] Rowida Baeshen et al. "Differential Effects of Inbreeding and Selection on Male Reproductive Phenotype Associated with the Colonization and Laboratory Maintenance of Anopheles Gambiae". In: *Malaria Journal* 13.1 (Jan. 2014), p. 19. ISSN: 1475-2875. DOI: 10.1186/1475-2875-13-19.

[53] Perran A. Ross, Nancy M. Endersby-Harshman, and Ary A. Hoffmann. "A Comprehensive Assessment of Inbreeding and Laboratory Adaptation in Aedes Aegypti Mosquitoes". In: *Evolutionary Applications* 12.3 (2019), pp. 572–586. ISSN: 1752-4571. DOI: 10.1111/eva.12740.

[54] Robert M May. *Stability and complexity in model ecosystems.* Princeton university press, 1973.

[55] Kym E Wilkins et al. "Pest demography critically determines the viability of synthetic gene drives for population control". In: *Mathematical Biosciences* 305 (2018), pp. 160–169.

[56] P. J. Beaghton and Austin Burt. "Gene Drives and Population Persistence vs Elimination: The Impact of Spatial Structure and Inbreeding at Low Density". In: *Theoretical Population Biology* 145 (June 2022), pp. 109–125. ISSN: 0040-5809. DOI: 10.1016/j.tpb.2022.02.002.

[57] Samuel E Champer et al. "Anopheles Homing Suppression Drive Candidates Exhibit Unexpected Performance Differences in Simulations with Spatial Structure". In: *eLife* 11 (Oct. 2022). Ed. by George H Perry, Sebald ANR Verkuijl, and Jim Bull, e79121. ISSN: 2050-084X. DOI: 10.7554/eLife.79121.

[58] Akira Okubo and Smon A Levin. *Diffusion and ecological problems: modern perspectives.* Vol. 14. Springer Science & Business Media, 2002.

[59] James D Murray. *Mathematical biology: I. An introduction.* Vol. 17. Springer Science & Business Media, 2007.

[60] WH Press et al. *Numerical Recipes in Fortran 77, The Art of Scientific Computing.*

[61] D. G. Aronson and Hans F Weinberger. "Multidimensional Nonlinear Diffusion Arising in Population Genetics". In: *Advances in Mathematics* 30.1 (Oct. 1978), pp. 33–76. ISSN: 0001-8708. DOI: 10.1016/0001-8708(78)90130-5.

[62] Frederik J. H. de Haas et al. "Modelling daisy quorum drive: A short-term bridge across engineered fitness valleys". In: *PLOS Genetics* 20.5 (May 2024), pp. 1–26. DOI: 10.1371/journal.pgen.1011262.

[63] Ace North, Austin Burt, and H. Charles J. Godfray. "Modelling the Spatial Spread of a Homing Endonuclease Gene in a Mosquito Population". In: *Journal of Applied Ecology* 50.5 (2013), pp. 1216–1225. ISSN: 1365-2664. DOI: 10.1111/1365-2664.12133.

[64] Philip A. Eckhoff et al. "Impact of Mosquito Gene Drive on Malaria Elimination in a Computational Model with Explicit Spatial and Temporal Dynamics". In: *Proceedings of the National Academy of Sciences* 114.2 (Jan. 2017), E255–E264. DOI: 10.1073/pnas.1611064114.

[65] Richard Durrett and Simon Levin. "The importance of being discrete (and spatial)". In: *Theoretical population biology* 46.3 (1994), pp. 363–394.

[66] Léna Kläy et al. "Stochastic dynamics at the back of a gene drive propagation wave". In: *arXiv preprint arXiv:2502.21268* (2025).

[67] Keith D Harris and Gili Greenbaum. "Rescue by gene swamping as a gene drive deployment strategy". In: *Cell Reports* 42.12 (2023).

[68] Jaroslav Piálek and Nick H Barton. "The spread of an advantageous allele across a barrier: the effects of random drift and selection against heterozygotes". In: *Genetics* 145.2 (1997), pp. 493–504.

[69] Timothy H Keitt, Mark A Lewis, and Robert D Holt. "Allee effects, invasion pinning, and species' borders". In: *The American Naturalist* 157.2 (2001), pp. 203–216.

[70] Andrea K. Beaghton et al. "Gene Drive for Population Genetic Control: Non-Functional Resistance and Parental Effects". In: *Proceedings of the Royal Society B: Biological Sciences* 286.1914 (Oct. 2019), p. 20191586. DOI: 10.1098/rspb.2019.1586.

[71] Andrew M. Hammond et al. "The Creation and Selection of Mutations Resistant to a Gene Drive over Multiple Generations in the Malaria Mosquito". In: *PLOS Genetics* 13.10 (Oct. 2017), e1007039. ISSN: 1553-7404. DOI: 10.1371/journal.pgen.1007039.

[72] Tom A. R. Price et al. "Resistance to Natural and Synthetic Gene Drive Systems". In: *Journal of Evolutionary Biology* 33.10 (Oct. 2020), pp. 1345–1360. ISSN: 1010-061X. DOI: 10.1111/jeb.13693.

[73] Loïc Marrec and Anne-Florence Bitbol. "Adapt or perish: evolutionary rescue in a gradually deteriorating environment". In: *Genetics* 216.2 (2020), pp. 573–583.

[74] Andri Manser et al. "Controlling Invasive Rodents via Synthetic Gene Drive and the Role of Polyandry". In: *Proceedings of the Royal Society B: Biological Sciences* 286.1909 (Aug. 2019), p. 20190852. DOI: 10.1098/rspb.2019.0852.

[75] Andri Manser, Barbara König, and Anna K. Lindholm. "Polyandry Blocks Gene Drive in a Wild House Mouse Population". In: *Nature Communications* 11.1 (Nov. 2020), p. 5590. ISSN: 2041-1723. DOI: 10.1038/s41467-020-18967-8.

[76] Aysegul Birand et al. "Gene Drives for Vertebrate Pest Control: Realistic Spatial Modelling of Eradication Probabilities and Times for Island Mouse Populations". In: *Molecular Ecology* 31.6 (Mar. 2022), pp. 1907–1923. ISSN: 0962-1083. DOI: 10.1111/mec.16361.

[77] John M. Marshall and Bruce A. Hay. “Medusa: A Novel Gene Drive System for Confined Suppression of Insect Populations”. In: *PLOS ONE* 9.7 (July 2014), e102694. ISSN: 1932-6203. DOI: 10.1371/journal.pone.0102694.

[78] Héctor M. Sánchez C. et al. “MGDrivE: A Modular Simulation Framework for the Spread of Gene Drives through Spatially Explicit Mosquito Populations”. In: *Methods in Ecology and Evolution* 11.2 (2020), pp. 229–239. ISSN: 2041-210X. DOI: 10.1111/2041-210X.13318.

[79] Samuel E. Champer et al. *Finding the Strongest Gene Drive: Simulations Reveal Unexpected Performance Differences between Anopheles Homing Suppression Drive Candidates*. Mar. 2022. DOI: 10.1101/2022.03.28.486009.

[80] Yiran Liu et al. “Adversarial Interspecies Relationships Facilitate Population Suppression by Gene Drive in Spatially Explicit Models”. In: *Ecology Letters* 26.7 (2023), pp. 1174–1185. ISSN: 1461-0248. DOI: 10.1111/ele.14232.

[81] Jun Li et al. “Can CRISPR Gene Drive Work in Pest and Beneficial Haplodiploid Species?” In: *Evolutionary Applications* 13.9 (2020), pp. 2392–2403. ISSN: 1752-4571. DOI: 10.1111/eva.13032.

[82] John B. Connolly et al. “Gene Drive in Species Complexes: Defining Target Organisms”. In: *Trends in Biotechnology* 41.2 (Feb. 2023), pp. 154–164. ISSN: 0167-7799. DOI: 10.1016/j.tibtech.2022.06.013.

[83] National Academies of Sciences, Engineering, and Medicine. *Gene Drives on the Horizon: Advancing Science, Navigating Uncertainty, and Aligning Research with Public Values*. Washington (DC): National Academies Press (US), 2016. ISBN: 978-0-309-43787-5.

[84] Martin Strugarek and Nicolas Vauchelet. “Reduction to a Single Closed Equation for 2-by-2 Reaction-Diffusion Systems of Lotka–Volterra Type”. In: *SIAM Journal on Applied Mathematics* 76.5 (Jan. 2016), pp. 2060–2080. ISSN: 0036-1399. DOI: 10.1137/16M1059217.
